# Cytolytic activity correlates with the mutational burden and deregulated expression of immune checkpoints in colorectal cancer

**DOI:** 10.1186/s13046-019-1372-z

**Published:** 2019-08-20

**Authors:** Apostolos Zaravinos, Constantinos Roufas, Majdi Nagara, Beatriz de Lucas Moreno, Maria Oblovatskaya, Christodoulos Efstathiades, Christos Dimopoulos, Georgios D. Ayiomamitis

**Affiliations:** 1 Department of Life Sciences, School of Sciences, European University Cyprus, 1516 Nicosia, Cyprus; 2 Centre for Risk and Decision Sciences (CERIDES), 2404 Nicosia, Cyprus; 3 Department of Computer Science & Engineering, European University Cyprus, 1516 Nicosia, Cyprus; 4 Inserm, UMR-S 1251, MMG, Faculté de Médecine, Aix Marseille University, Marseille, France; 5 Center for Research in Health and Life Sciences, European University Madrid, 28670 Madrid, Spain; 6 1st Surgical Department, Tzaneion General Hospital, 18536 Piraeus, Greece

**Keywords:** Colorectal cancer, Cytolytic activity, Classically defined neoepitopes, Alternatively defined neoepitopes, Mutation burden, Immune checkpoints, Cancer drivers

## Abstract

**Background:**

Microsatellite unstable colorectal cancers (MSI+ CRCs) expressing PD-L1, respond to anti-PD-1 or anti-PD-L1 checkpoint blockade, whereas microsatellite-stable tumors do not respond the same. Our aim was to examine how the immune landscape relates to different aspects of the CRC’s biology, including neoepitope burden.

**Methods:**

We used TCGA data to stratify patients based on a cytolytic T-cell activity expression index and correlated immune cytolytic activity (CYT) with mutational, structural, and neoepitope features of each tumor sample. The expression of several immune checkpoints was verified in an independent cohort of 72 CRC patients, relative to their MSI status, using immunohistochemistry and RT-qPCR.

**Results:**

CRC exhibits a range of intertumoral cytolytic T-cell activity, with lower cytolytic levels in the tumor, compared to the normal tissue. We separated CRC patients into CYT-high and CYT-low subgroups. High cytolytic activity correlated with increased mutational load in colon tumors, the count of MHC-I/−II classically defined and alternatively defined neoepitopes, high microsatellite instability and deregulated expression of several inhibitory immune checkpoints (VISTA, TIGIT, PD-1, IDO1, CTLA-4, and PD-L1, among others). Many immune checkpoint molecules (IDO1, LAG3, TIGIT, VISTA, PD-1, PD-L1 and CTLA-4) expressed significantly higher in MSI+ CRCs compared to MSS tumors. The expression of Treg markers was also significantly higher in CYT-high tumors. Both individual and simultaneous high levels of CTLA-4 and PD-L1 had a positive effect on the patients’ overall survival. On the reverse, simultaneous low expression of both genes led to a significant shift towards negative effect. Assessed globally, CYT-low CRCs contained more recurrent somatic copy number alterations. PD-L1 protein was absent in most samples in the independent cohort and stained lowly in 33% of MSI CRCs. PD-L1+ CRCs stained moderately for CD8 and weakly for FOXP3. CYT-high colon tumors had higher TIL load, whereas CYT-high rectum tumors had higher TAN load compared to their CYT-low counterparts.

**Conclusions:**

Overall, we highlight the link between different genetic events and the immune microenvironment in CRC, taking into consideration the status of microsatellite instability. Our data provide further evidence that MSI+ and CYT-high tumors are better candidates for combinatorial checkpoint inhibition.

**Electronic supplementary material:**

The online version of this article (10.1186/s13046-019-1372-z) contains supplementary material, which is available to authorized users.

## Background

Colorectal cancer (CRC) is the second leading cause of cancer-related death with > 8% of the newly estimated cases and deaths [1]. The introduction of EGFR and VEGF targeted agents to standard chemotherapy has brought modest advances in the treatment against the disease, prolonging overall survival up to 30 months in patients with metastatic disease [2]. However, recent trial results on PD1 and PD-L1 blockade provide encouraging evidence, that immunotherapy can further improve the therapeutic path.

A progressive accumulation of genetic and epigenetic events in tumor suppressor genes (*APC*, *TP53*, *SMAD4*) and oncogenes (*KRAS*, *PI3K, BRAF*) leads to the progression of adenoma to carcinoma. The majority of CRCs (~ 85%) develop because of chromosomal instability (CIN), loss of heterozygosity (LOH), chromosomal amplifications and translocations; while the remaining 15% have defective DNA mismatch repair systems (MMR) caused by the inactivation in *MLH1*, *MLH3*, *MSH2*, *MSH3*, *MSH6*, or *PMS2*, leading to hypermutations and microsatellite instability (MSI) [3]. Eventually, the accumulation of such DNA mutations promotes the formation of immunogenic tumor-mutated peptides called neoantigens (or neoepitopes), which attract a high number of tumor-infiltrating lymphocytes (TILs) and other immune cells into the tumor’s microenvironment [4]. High numbers of CD8+ TILs are good indications of response to neoadjuvant chemotherapy [5]. Consequently, the tumor’s biology is to a significant extent regulated by immune cells within the tumor’s microenvironment. The patient’s antitumoral immune cytolytic activity (CYT), calculated as the geometric mean of the expression of the genes granzyme A (GZMA) and perforin 1 (PRF1), is also associated with improved patient survival [6, 7]. Cytotoxic T cells (CTL) and natural killer cells (NK) are able to kill tumor cells by overexpressing GZMA and PRF1 [8]. It is also known that effector T cells at the tumor site can predict a favorable outcome across many cancers [9–14]. GZMA is a tryptase that leads to caspase-independent apoptosis, while PRF1 is a pore-forming enzyme that facilitates the entry of granzymes into the target cells. Both effector molecules are considerably over-expressed upon CD8+ T cell activation [15] and during productive clinical responses to anti-CTLA-4 or anti-PD-L1 treatment [16, 17].

Recently, CRC was divided to four consensus molecular subtypes (CMSs) which can be used to classify individual patients who have higher chances to respond to targeted therapies. These include CMS1 (microsatellite instability immune, 14%), CMS2 (canonical, 37%), CMS3 (metabolic, 13%), and CMS4 (mesenchymal, 23%) [18]. CMS1 subtype tumors have the potential to generate durable clinical responses [19]. Immunologically, the microenvironment of colorectal cancers is rich in immunosuppressive cytokines. It is also characterised by inhibition of T-cell proliferation and effector responses, as well as tissue hypoxia [20]. To date, there is provocative evidence that the levels of cytotoxic T cells significantly affect the overall survival of CRC patients [6].

Microsatellite unstable (MSI-H) tumors are also hypothesized to have a noteworthy immunological response, being stimulated by neoepitopes that are created due to a defective MMR. Several immune checkpoint inhibitors, including anti-PD-1 or anti-PD-L1 monoclonal antibodies, are currently being clinically tested or used to therapeutically treat different cancer types, including metastatic colorectal cancer [21–26].

Herein, we investigated whether CYT is associated with distinct mutational and expressional profiles, as well as with the individual tumor’s neoepitope load in CRC. To this end, we examined how the immune landscape of colorectal cancer relates to different aspects of the tumor’s biology, including microsatellite instability, somatic mutations and copy number aberrations, the expression of immune checkpoint molecules and TIL load, or the presence of other immune cells.

## Methods

### Extraction of colorectal cancer datasets

Colorectal cancer data were extracted from The Cancer Genome Atlas (TCGA) datasets TCGA-COAD (colon adenocarcinoma, *n* = 480) and TCGA-READ (rectum adenocarcinoma, *n* = 367) and represent only untreated primary tumors. Patients who received neo-adjuvant therapy were not included in the study. The exact tumor samples within each dataset, along with their clinical information and cytolytic levels (CYT), are noted in Additional file 10: Table S1. Patients in both datasets were stratified to microsatellite unstable (MSI) or stable (MSS) according to the presence of missense mutations detected in the MMR genes *MLH1*, *MLH3*, *MSH2*, *MSH3*, *MSH4*, *MSH5*, *MSH6*, *PMS1* and *PMS2* (Additional file 11: Table S2).

“Level 3” gene expression data, mutational annotation format (MAF) files, copy number variation (CNV) files, and each patient’s clinical information, were all extracted from TCGA’s public access Genomic Data Commons data portal (https://portal.gdc.cancer.gov/). GISTIC2.0 [27] gene-level, zero-centered, focal copy number calls for each CRC patient were accessed from Broad Institute’s GDAC Firehose (https://gdac.broadinstitute.org/). Data were pre-processed in Apache Spark and further analyzed using the R environment.

### Calculation of cytolytic activity

We calculated the immune cytolytic activity as the geometric mean of the genes *GZMA* and *PRF1*, as previously mentioned [7, 28]. Briefly, we divided each gene’s total raw read counts by its maximum transcript length to represent a coverage depth estimate. Coverage estimates were then scaled to sum to a total depth of 1e6 per sample and deduced as transcripts per million (TPM), after adding a 0.01 offset to remove the zero counts from calculations.

COAD and READ dataset patients were separated into a CYT-high cohort (upper 25th quartile cytolytic index) and a CYT-low cohort (lower 25th quartile cytolytic index), each with equal combinations of histology-stage mixture. Comparisons were made between CYT-high or -low COAD and READ cancers, respectively. The *p*-values from the comparisons of the cytolytic activity between samples were FDR-adjusted.

### RNA-seq-based gene expression and protein expression analysis

We performed gene set variation analysis (GSVA) to subtle pathway activity changes over the sample population and to estimate variation of gene set enrichment across each dataset, using the “GSVA” (v.1.23.4) R package. The sample-wise enrichment score for a given gene set was calculated using a Kolmogorov–Smirnov (KS)-like random walk statistic. Gene sets were extracted from the C2 collection of the Molecular Signatures Database (MSigDB). Statistical ranking for GSVA scores for the cytolytic index by the top and bottom quartiles were termed “CYT-high” and “CYT-low”, respectively. Unsupervised hierarchical clustering, using complete linkage with the distance metric equal to 1-Pearson’s correlation coefficient, was also executed using the GSVA scores for the COAD and READ datasets.

Differential gene expression analysis between CYT-high and CYT-low across each dataset was calculated using gene-level raw counts with the “limma” R package and voom transformation with quantile normalization (Additional file 1: Figure S1).

Lowly expressed genes with < 1 CPM in < 50% of the samples within each dataset were excluded for differential gene expression analysis. Genes with an FDR-adjusted *p*-value< 0.1 were considered as differentially expressed. All graphs were produced using the “ggplot2” R package.

GZMA and PRF1 immunohistochemistry (IHC) protein expression data were retrieved from the Human Protein Atlas (HPA) [29] and further analyzed.

### Analysis of somatic mutations and copy number alterations per cytolytic subset

We processed TCGA-extracted MAF files using the R/Bioconductor package “Maftools” and compared each cytolytic subgroup against the load of somatic mutations, microsatellite instability (MSI) and copy number alterations (CNAs). Adjusted *p*-values with statistical significance at adj. *p* < 0.01 was used to account for multiple testing.

Significantly mutated genes (SMG, FDR < 0.1) in cytolytic subtypes of colon and rectal adenocarcinomas were calculated using the MutSig algorithm (v1.3.01). Non-silent point mutations among the SMGs were investigated for association with each cytolytic subgroup, using a regression-based approach, as previously described in detail [30]. Mutually exclusive or co-occurring gene sets were detected using a pair-wise Fisher’s exact test.

GISTIC (v2.0.22) was used to detect recurrent somatic CNAs (SCNA) in CYT-high and -low colon and rectum cancers, respectively, using MutSigCV (v1) using the Broad Institute’s GenePattern. Each SCNA was assigned a G-score indicative of its amplitude and occurrence across samples. SCNAs in each CRC sample within each cytolytic subgroup were counted by taking the sum of segment mean changes ≥0.6 and ≤ − 0.4 between somatic and normal samples. Significantly amplified or deleted genomic regions in each cytolytic subgroup with FDR < 0.25 were considered significant.

### Tumor heterogeneity and MATH scores

Tumor heterogeneity in the COAD and READ datasets was inferred by clustering variant allele frequencies (VAF). The extent of intra-tumor heterogeneity of each tumor was quantitatively measured calculating the width of the VAF distribution and assigning a mutant-allele tumor heterogeneity (MATH) score, as the ratio of the width to the center of its distribution of mutant-allele fractions among tumor-specific mutated loci. No significant differences were scored between the two cytolytic subsets in each dataset.

### Overall survival and synergistic target analysis

We performed Kaplan-Meier survival analysis using the log-rank (Mantel Cox) test in each cytolytic CRC subgroup, with a *p* = 0.05 as threshold of statistical significance. The synergistic effect of the cytolytic genes *PRF1* and *GZMA* on patient survival was further tested using SynTarget [31].

### Intratumoral immune cell composition

We used the CIBERSORT [32] deconvolution algorithm (https://cibersort.stanford.edu/) to estimate the abundance of 22 immune cell types in each cytolytic subgroup’s tissue and to evaluate the corresponding intratumoral immune cell composition.

### Neoepitope analysis

The “antigen.garnish” R package was used to predict neoepitopes from different DNA variants (missense mutations, indels and gene fusions) that were found across CYT-high and –low CRCs. Peptides (mutant nmers) predicted to bind MHC with high affinity (IC_50_ < 50 nM) or with greatly improved affinity relative to their non-mutated counterparts (differential agretopicity index (DAI) > 10 for MHC-I and > 4 for MHC-II), were classified as classically defined neoepitopes (CDNs) or alternatively defined neoepitopes (ADNs), respectively. Mutant peptides that met both ADN and CDN criteria, or those that met the CDN criteria and were derived from frameshift mutations, were defined as priority neoepitopes. The load of cancer neoepitopes was associated with the CYT and MATH scores in each dataset, using Pearson’s correlation.

### Validation of gene expression in an independent cohort of colorectal cancer samples

Seventy-two colorectal cancer tissue samples were surgically extracted at the Tzaneion General Hospital, Piraeus. Directly after resection, the samples were stored at − 80 °C in RNA*later*. Histological classification was implemented according to the WHO and staging according to the UICC-TNM classification (2002). Informed consent was obtained from all patients and the study protocol was approved by the Hospital’s Ethics Committee (TGH#16527/4-12-2017). A matched normal mucosa biopsy was also collected from each patient. Samples were homogenized in Trizol and total RNA was extracted and further purified using RNeasy kit (Qiagen) after on-column DNAse digestion, prior to reverse transcription and real-time PCR (RT-qPCR). cDNA was synthesized using the qScript system (Quanta Biosciences, Gaithersburg, MD). Real-time PCR was performed using a CFX96 Touch™ Real-Time PCR Detection System (Bio-Rad) with 2x Kapa SYBR Fast qPCR Master Mix Universal (Sigma-Aldrich) in triplicate 20 μl reactions and analyzed on the CFX Manager (Bio-Rad). Relative quantification of each target’s mRNA levels was performed using the Pfaffl method [33]. Primer sequences derived from the PrimerBank [34] (Additional file 12: Table S3).

All tumor samples were divided to high- or low- frequency microsatellite unstable (MSI-H, *n* = 12 and MSI-L, *n* = 13, respectively) and microsatellite stable (MSS, *n* = 47) tumors, using a panel of three dinucleotide repeat markers (D2S123, D5S346, D17S250) and two mononucleotide repeat markers (BAT25 and BAT26), as previously defined [35].

### Immunohistochemistry (IHC) and TIL evaluation

Tissue samples from the same cohort of patients were formalin-fixed and paraffin-embedded (FFPE). Each paraffin section was reviewed by a pathologist, histologically classified according to the WHO and staged according to the UICC-TNM classification (2009).

IHC analysis for the protein expression of MLH1, MSH2, PD-L1, CD8, FOXP3 and CD66b was done as previously described [36, 37]. Briefly, one block of FFPE tumor tissue, usually comprising adjacent normal mucosa, was selected per case. Five-μm-thick sections were deparaffinized and rehydrated using xylene and alcohol. Endogenous peroxidase was blocked with H_2_O_2_ 0.3% in Tris buffer (pH 7.6) for 15 min. Before immunostaining, the sections were immersed in 10 mM citrate buffer (pH 6.0), rinsed in Tris-buffered saline (TBS) and subjected to heat-induced antigen retrieval in a microwave oven (30 min at 600 W). Sections were then incubated overnight at 4°C with mouse monoclonal antibodies against PDL1 (1:50 dilution, clone 22C3, Dako, CA), CD8 (1:400 dilution, clone C8/144B, Dako, CA), FOXP3 (1:200 dilution, clone 236A/E7, ThermoFischer Scientific), CD66b (1:200 dilution, clone G10F5, BD Biosciences), MLH1 (1:100 dilution, clone E505, Dako, CA), and MSH2 proteins (1:100 dilution, clone FE11; Oncogene Research Products, MA). The EnVision™ FLEX+ Mouse (linker) detection kit was used as the secondary detection system, and the peroxidase reaction was developed using 3,3′-DAB. Subsequently, slides were washed thoroughly in running tap water and counterstained with hematoxylin before being dehydrated and mounted.

Hyper-reactive tonsil sections were used as positive controls for anti-PDL1 and anti-CD8 staining. Preimmune rabbit serum was used as a negative control to test for nonspecific staining. Two pathologists, blinded to clinical information, independently evaluated immunoreactivity by assessing the percentage of positively immunostained tumor cells. Discrepancies were resolved by consensus. MLH1 and MSH2 protein expression was scored as positive if 10% of cells were found positive.

MMR protein loss was defined as the absence of nuclear staining in tumor cells in the presence of positive nuclear staining in normal epithelial cells and lymphocytes. Tumors were categorized as having deficient MMR (dMMR/MSI) if the expression of at least one protein was lost, and proficient MMR (pMMR/MSS) if all proteins were intact.

CD8 is specific for cytotoxic T-cells, with a low percentage (< 25%) of them being negatively stained in the cytoplasm/membrane. FOXP3 is a marker for Treg cells, and exhibited very low nucleoplasmic staining. PD-L1 a marker specific for T-cells, B-cells and tumor cells, was mainly lowly expressed or absent (< 10%, membranous staining). CD66b, a marker specific for tumor-associated neutrophils (TAN), exhibited strong cytoplasmic/membranous staining in most cells (> 75%).

The stains for PD-L1, CD8, FOXP3 and CD66b were scored using the 0–2+ scale: 0 for no staining, 1+ for faint staining, and 2+ for moderate or strong staining. CRC patients were also separated into high- and low-TIL or TAN load groups, based on the median number of TILs (n=5%), or that of CD66b + TANs (n=2%).

Hematoxylin and eosin (H&E) sections were systematically reviewed for pathologic features including tumor histologic type, differentiation, TILs and peritumoral lymphocyte aggregates. TILs were scored from 0 to 3, with 0 being < 1 TIL, 1 being 1–15 TILs, 2 being > 15 but < 215 TILs, and 3 being > 215 TILs per 10 high power fields. Peritumoral lymphocyte aggregates were scored from 0 to 2 with 0 being none, 1 being a few, often < 5, and 2 being > 5. All tissue slides were scanned on a VENTANA iScan HT slide scanner v1.1.1 (Roche) and analyzed using the corresponding software.

## Results

### CRC stratification based on the immune cytolytic T-cell activity

To evaluate intertumoral immune cytolytic T-cell activity across the colon (COAD) and rectum (READ) adenocarcinoma samples, we initially calculated the transcript levels of GZMA and PRF1 [7, 28].

**Fig. 1:**
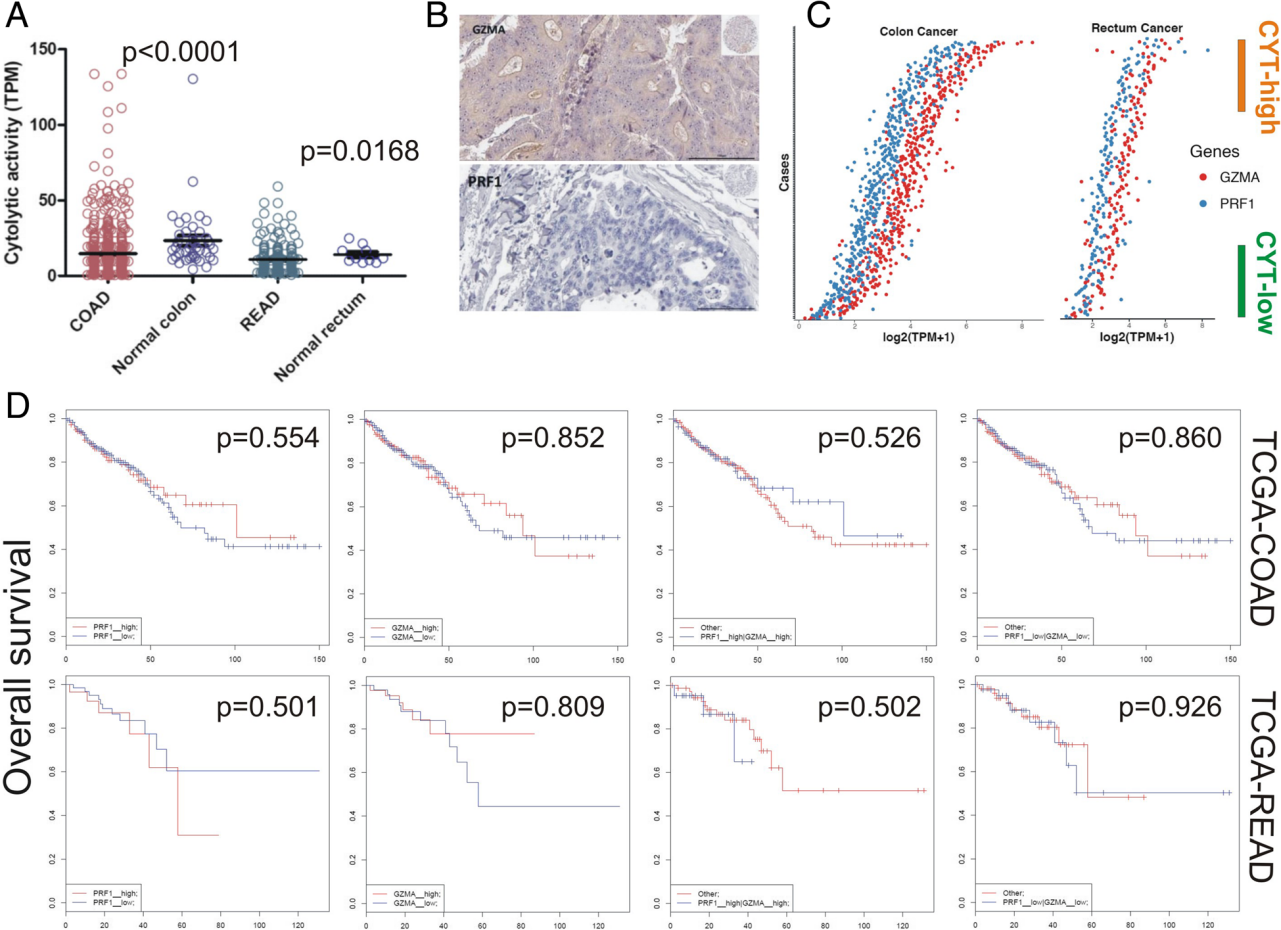
**a** CYT levels were significantly lower in the COAD and READ datasets compared to the normal colon and rectum, respectively. **b** GZMA and PRF1 proteins were lowly, or not expressed in immunohistochemistry (IHC) protein expression data retrieved from the Human Protein Atlas (HPA). The minimized upper right image depicts the whole tissue section in each tissue microarray (TMA) slide. **c** Distribution of cytolytic genes within COAD and READ tumors. Gene set variation analysis (GSVA) signature scores for cytolytic index distinguished top quartile (orange) and bottom quartile (green) samples for cytolytic-high (CYT-high) and low (CYT-low) tumors, respectively. **d** Kaplan-Meier curve analysis of GZMA and PRF1 shows that high expression of both GZMA and PRF1 in colon (but not rectal) cancers, synergistically affects the patients' overall survival

Cytolytic levels were significantly lower in both datasets compared to the normal colon and rectum, respectively (Fig. 1a). The results were further supported using HPA-derived protein expression data, in which GZMA was lowly expressed in 3/11 colorectal cancer samples, and not detected in 8/11 of them. On the other hand, PRF1 protein was not detected in any of the 12 colorectal cancer samples (Fig. 1b and Additional file 13: Table S4). To classify the CRC subpopulations according to high or low cytolytic activity, we stratified each CRC dataset by defining cancer samples in the top 25th percentile by cytolytic index (log_2_TPM + 1), as “CYT-high” and those in the bottom 25th percentile, as “CYT-low” (Fig. 1c and Additional file 14: Table S5). GZMA and PRF1 exhibited significantly coordinated roles, mainly in the COAD dataset (*r* = 0.677, *p* = 1.27E-06) (Fig. 1c).

In the COAD (but not READ) dataset, both individual and simultaneous high levels of *PRF1* and *GZMA* had a positive effect on the patients’ overall survival, according to SynTarget analysis. On the contrary, simultaneous low expression of both genes led to a significant shift towards negative effect versus all other patients, indicating the synergetic effect of both genes on patients’ survival outcome in CRC. (Fig. 1d).

### Cytolytic activity varies across different CRC subtypes

**Fig. 2:**
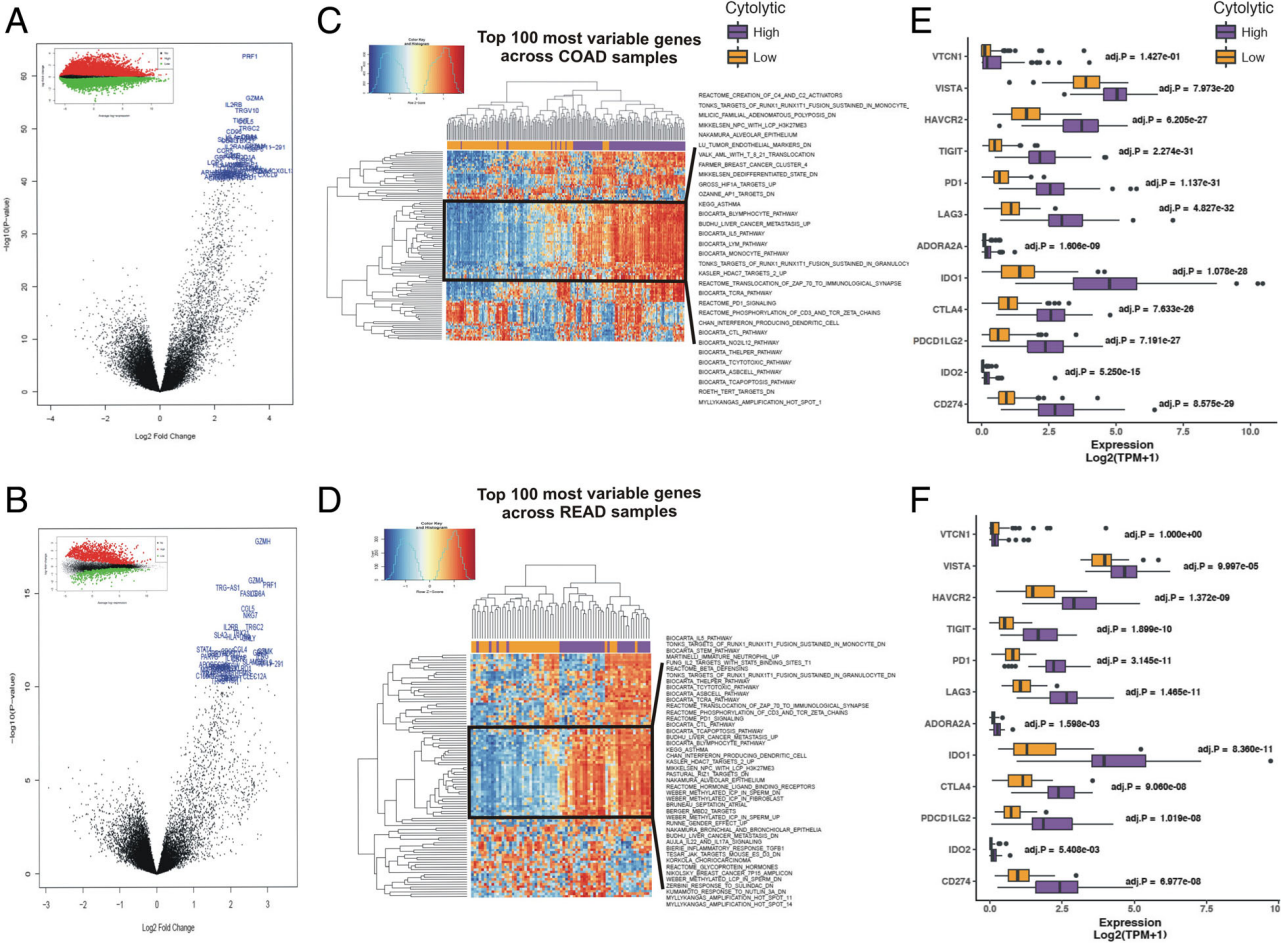
**a-b** Volcano plots for differential gene expression (average log fold change) in the two cytolytic subgroups of COAD (**a**) and READ (**b**) tumors. The top 100 significantly upregulated genes in CYT-high tumors are highlighted in blue. The mean-difference (MD) plots on top of each subfigure depict the up- (red) and down-regulated genes (green) in CYT-high vs -low tumors. **c-d** Two-way hierarchical clustering of differentially activated pathways at 0.1% false discovery rate (FDR) in the CYT-high COAD (**c**) and READ (**d**) tumors. Both datasets were statistically enriched for immune gene programs, which contain markers for T-cell inhibition and CD8+ T-cells and B-cells. **e-f** The expression of several inhibitory immune checkpoint molecules, including VTCN1, VISTA, HAVCR2, TIGIT, PD-1, LAG3, ADORA2A, IDO1, IDO2, CTLA-4, CD274 (PD-L1) and PDCD1LG2 (PD-L2) was significantly higher in the CYT-high immune cytolytic subgroups of the COAD (**e**) and READ (**f**) datasets

We then predicted the differentially expressed genes between the two immune cytolytic subgroups in each CRC dataset. As expected, *GZMA* and *PRF1* were among the top upregulated genes in the cytolytic-high tumors (Fig. 2a-b). Several other immune-related molecules, including *IL2RB*, *TRGV10*, *TIGIT*, *CCL5*, *TRGC2*, *CD96*, *HLA*-*DRA*, *CD8A*, *GZMH*, *TRG-AS1*, *FASLG*, and *NKG7* were included within the top-upregulated genes in the CYT-high subgroups in both datasets, clarifying their involvement in the tumor microenvironment (Additional file 15: Table S6).

Cytolytic-high CRC samples were enriched for gene sets associated with activated CD8+, PD1^high^ T-cells [38, 39], confirming that the expression of GZMA and PRF1 correlates with immune response and infiltration of CD8+ cytolytic T-cells (Additional file 2: Figure S2). These data suggest that the stratification based on cytolytic T-cell infiltration, as measured by the cytolytic index, may be associated with distinct CRC subtypes. We therefore, determined whether cytolytic activity is associated with genomic and transcriptional metrics of the biology of colon and rectal adenocarcinomas.

We assessed enrichment of gene sets defining colon and rectum adenocarcinoma subtypes, and investigated their association with the cytolytic index. CYT-high tumors were statistically enriched for immune gene sets containing Lck and Fyn, the responsible tyrosine kinases for the initiation of TCR activation, CD8+ T-cytotoxic cell, B-cell and T-helper cell surface molecules, CTL-mediated immune response genes against target cells, apoptosis, and genes involved in the IL-5 signaling pathway. CYT-high tumors were also enriched for genes being up-regulated in spleen interferon-producing dendritic cells (DCs) compared to plasmacytoid and conventional DCs, as well as in antigen-dependent B cell activation. CYT-high tumors were furher enriched in methylated germline-specific genes with intermediate-CpG-density promoters in sperm, amplification hotspots in loci 8q24.1-q24.3, 11q3, 18q11.2-q23 and Xp22.3-p11.1, neutrophil-specific genes up-regulated in comparison of immature with mature neutrophils, genes involved in PD-1 signaling, in the translocation of ZAP-70 to immunological synapse, and in the phosphorylation of CD3 and TCR zeta chains, among several other immune-related datasets (Fig. 2c-d and Additional file 3: Figure S3).

The expression of several immune checkpoints, including *VTCN1*, *VISTA*, *HAVCR2*, *IDO1*/2, *PD-1*, *PD-L1*, and *CTLA-4*, was also significantly elevated in the CYT-high subgroup of COAD and READ tumors (Fig. 2e-f). Importantly, high expression of *TIGIT*, *PD-1*, *LAG3*, *IDO1*, *CTLA-4*, *PDCD1LG2* (*PDL2*), *CD274* (*PD-L1*) and *HAVCR2*, was significantly correlated (*p* < 0.001, Pearson’s rho> 0.65) with an increased immune cytolytic activity in both CRC datasets. Similarly, but to a less extent, *VTCN1*, *VISTA*, *ADORA2A*, and *IDO2* correlated with high cytolytic levels (*p* < 0.01, Pearson's rho > 0.3)  (Fig. 4i-j). These data suggest that stratification of CRC patients based on transcriptional profiling can differentiate tumors with a strong cytolytic T-cell response, from those having an immune microenvironment that impedes such responses, thus, enhancing targeting of the tumor microenvironment [40, 41].

### Cytolytic activity correlates with distinct mutational events in CRC

**Fig. 3:**
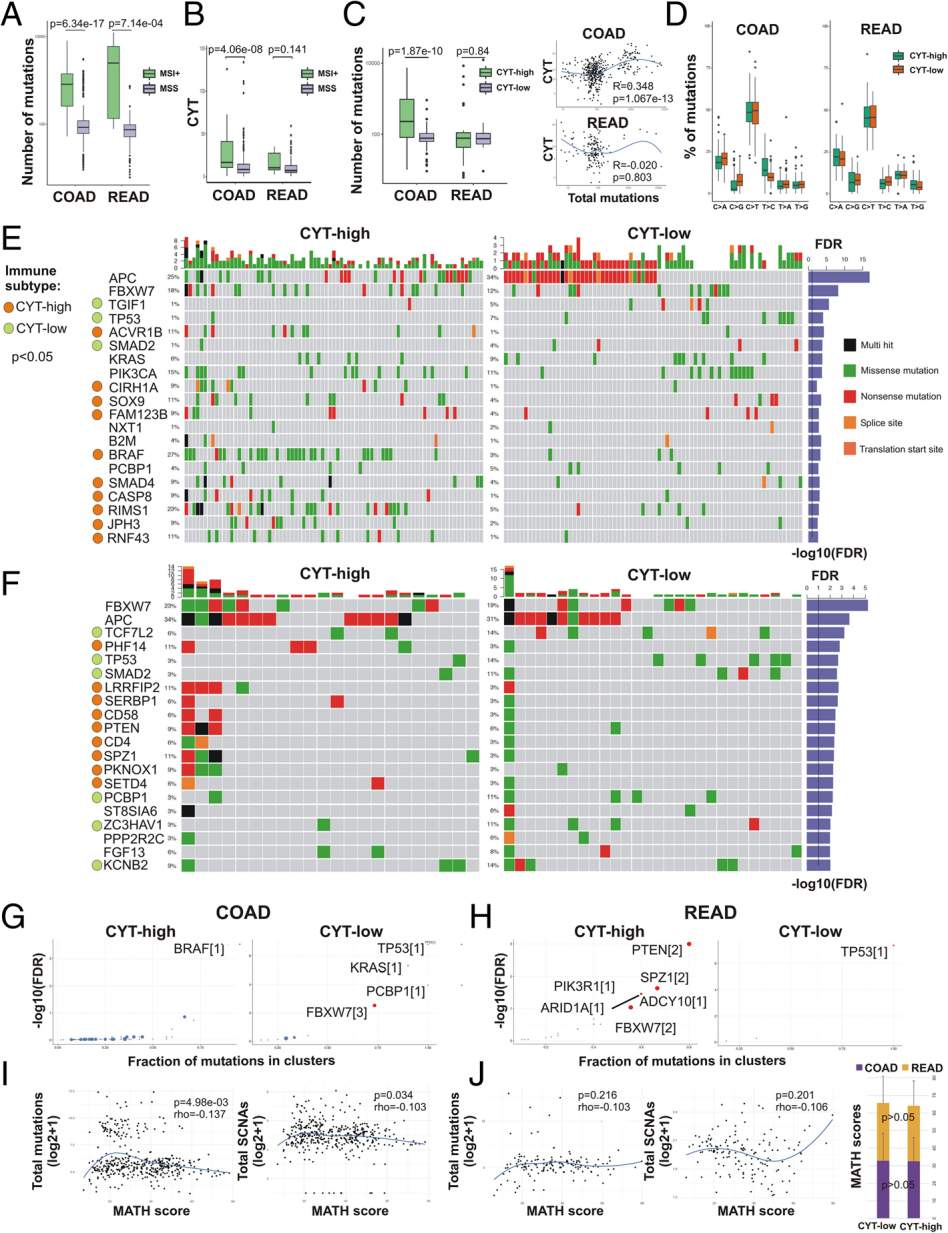
**a** The mutation load increased considerably in colorectal tumors with high microsatellite instability (MSI) vs stable microsatellites (MSS). **b** Cytolytic activity (CYT) is considerably higher in colon (but not rectal) tumors with high microsatellite instability (MSI+) vs MSS tumors. **c** The mutation load increased considerably in CYT-high colon (but not rectal) tumors and was significantly correlated with the cytolytic index (CYT). **d** Nonsynonymous mutation spectra across COAD and READ cytolytic subsets, depicting the percentage (%) of each mutation type in high and low colon and rectal tumors, respectively. Most mutations across the datasets were associated with C > T (and G > A) transitions and the frequency of specific substitutions did not differ between CYT-high and CYT-low tumors. **e-f** Co-mutation plot showing significantly mutated genes (SMGs, FDR < 0.1) in cytolytic subsets of colon (COAD) (**e**) and rectal (READ) tumors (**f**). Green, red, pink, black and orange boxes indicate missense, nonsense, transcription start site, multi-hit and splice-site mutations, respectively. SMGs that correlate with immune cytolytic subtypes (*p* < 0.05) are highlighted by green or orange circles in the left columns of each dataset. Each SMG’s q-values (−log_10_(FDR)) are plotted as a right-side bar plot in blue color. **g-h** Plots show the different cancer driver genes in the two cytolytic subgroups of COAD and READ adenocarcinomas, using OncodriveCLUST [42]. Cancer driver genes are depicted as scatter plots, in which the size of the points is proportional to the number of clusters found in the corresponding gene. The x-axis shows the fraction of mutations observed in these clusters. Scores within brackets next to the gene names denote the number of the gene’s mutational clusters. **i-j** Tumor heterogeneity and mutation load in CRC. The width of each tumor’s variant allele frequency (VAF) distribution (MATH scores) does not correlate with the total mutation count or the total copy number events, neither in colon (**i**) nor in rectal cancers (**j**). No difference in MATH scores between cytolytic subsets in COAD and READ cohorts (*p* > 0.05). *P*-values were calculated using Kruskal-Wallis test (**j**)

We next sought to determine whether CYT correlates with distinct mutational profiles characterized for CRC [3]. As expected, the mutation load increased significantly in microsatellite unstable (MSI+) colorectal tumors (Fig. 3a). Confirming previous findings [7], we found that cytolytic activity also increased dramatically given high MSI in colon (but not rectal) tumors (Fig. 3b). Furthermore, the mutation load increased considerably in CYT-high colon (but nor rectal) tumors (Fig. 3c). Most mutations associated with C > T transitions, without significant differences between CYT-high and -low tumors (Fig. 3d). Likewise, there was no association between the cytolytic index and *APC* or *KRAS* mutation types in either dataset (Additional file 4: Figure S4).

Motivated by these observations, we obtained curated mutational data for the two TCGA datasets and identified the significantly mutated genes (SMGs, FDR < 0.1) occurring in CYT-high and -low colon and rectal tumors, respectively. CYT-high colon cancers had a significant association (*p* < 0.05) with mutations in *ACVR1B*, *CIRH1A*, *SOX9*, *FAM123B*, *BRAF*, *SMAD4*, *CASP8*, *RIMS1*, *JPH3* and *RNF43*. On the other hand, CYT-low colon tumors were associated with missense, nonsense or splice-site mutations in *APC*, *TGIF1*, *TP53* and *SMAD2*. Furthermore, *FBXW7*, *KRAS*, *PIK3CA*, *NXT1*, *B2M* and *PCBP1* had equal mutation rates among CYT-high and -low colon cancers (Fig. 3e). Similarly, CYT-high rectal cancers correlated with mutations in *PHF14*, *LRRFIP2*, *SERBP1*, *CD58*, *PTEN*, *CD4*, *SPZ1*, *PKNOX1* and *SETD4*, and CYT-low rectum tumors were associated with missense, nonsense or splice-site mutations in *TCF7L2*, *TP53*, *SMAD2*, *PCBP1*, *ZC3HAV1* and *KCNB2*. Significantly mutated genes (FDR < 0.1) including *APC*, *FBXW7*, *ST8SIA6*, *PPP2R2C* and *FGF13* were detected in equal frequencies between both cytolytic subgroups of the READ dataset (Fig. 3f). Collectively, these data clarify an association of the cytolytic index with distinct somatic mutations in colorectal cancers.

We then hypothesized that different cancer driver genes are associated with each cytolytic subgroup in each dataset. To verify this assumption, we conducted OncodriveCLUST analysis [42] and identified a single significant *BRAF* mutation cluster among CYT-high colon tumors; whereas, in the CYT-low subgroup we detected different drivers, including *TP53*, *KRAS*, *PCBP1* (all of them with one mutation cluster) and *FBXW7*, with three mutation clusters (Fig. 3g). In CYT-high rectal cancers we also detected different cancer drivers between the two cytolytic subgroups. These, included *PTEN*, *SPZ1*, *FBXW7* (with two mutation clusters each) and *ADCY10*, *PIK3R1* and *ARIDA* (with one cluster each) in CYT-high rectum tumors; whereas in the CYT-low subgroup, *TP53* was the only cancer driver with one mutation cluster (Fig. 3h). Our data suggest that *TP53*, whose role as a cancer driver is well-known in colorectal cancer [43], is a common driver between CYT-high and –low tumors. They also show that apart from *TP53*, different cancer drivers exist within each cytolytic subgroup of colorectal tumors.

We further investigated mutually exclusive or co-occurring gene sets in each dataset, and found that *BRAF* was mutually exclusive with *APC* in COAD, while a broad combination of significant co-occurrences was scored between other gene pairs in both datasets, including *FBXW7* and *MUC16* (Additional file 5: Figure S5).

**Fig. 4:**
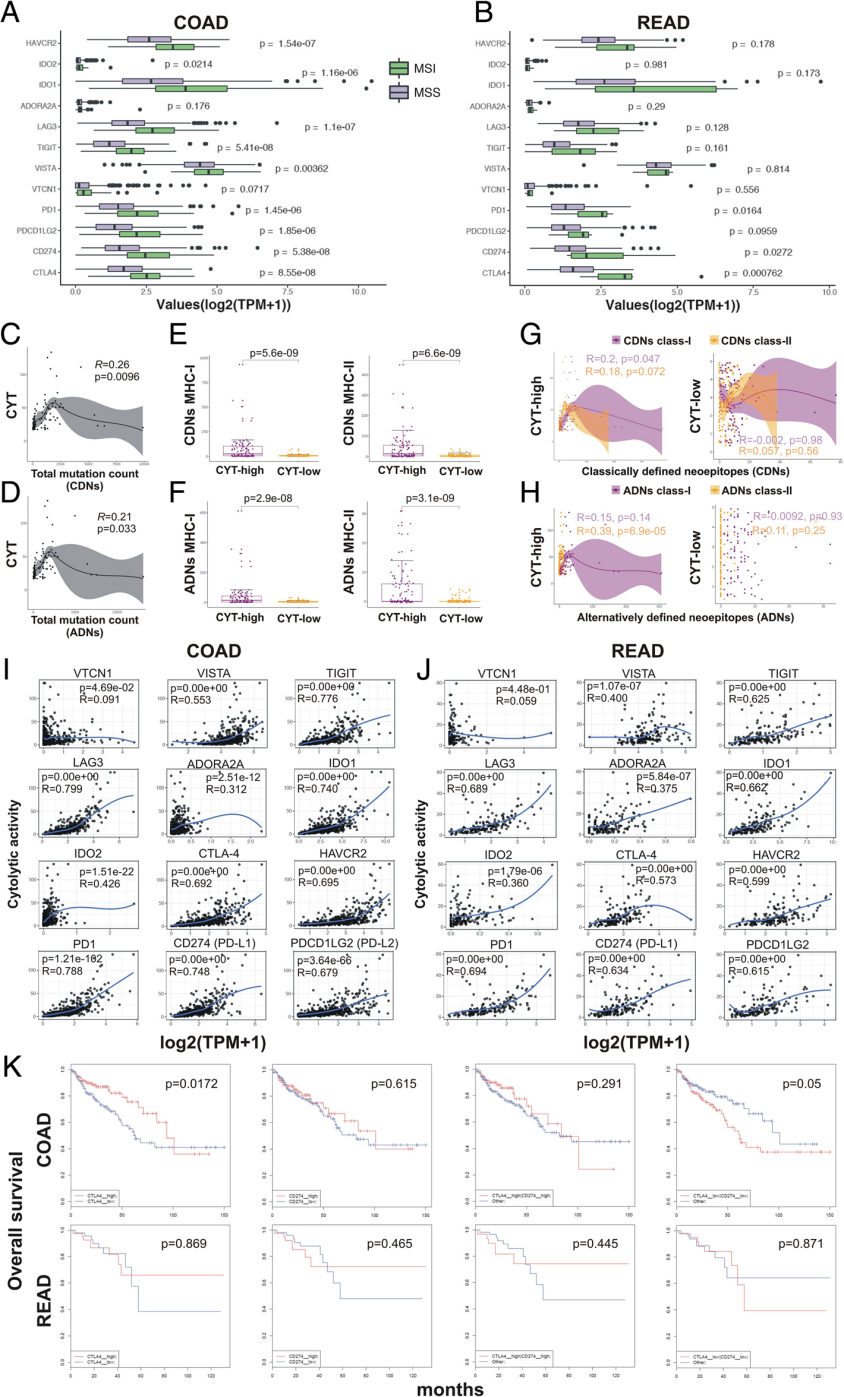
**a-b** The immune checkpoint molecules HAVCR2, IDO1, IDO2, ADORA2A, LAG3, TIGIT, VISTA, VTCN1, PD-1, PDCD1LG2 (PD-L2), CD274 (PD-L1) and CTLA-4 are significantly higher in MSI colorectal adenocarcinomas, compared to their MSS counterparts. **c-d** The total count of classically defined neoepitopes (CDNs) and alternatively defined neoepitopes (ADNs) per individual COAD tumor was significantly correlated with the levels of cytolytic activity (CYT). **e-f** The number of MHC class I and II CDN and ADN was significantly higher in CYT-high COAD tumors. **g-h** High levels of cytolytic activity (CYT-high) were significantly correlated with MHC-I CDNs (*p* = 0.047, Pearson’s rho = 0.2) and with MHC-II ADNs (*p* = 6.9e-05, Pearson’s rho = 0.39) in COAD tumors. **i-j** Pairwise correlation between the cytolytic index (log-average of GZMA and PRF1) versus individual genes of immune suppression index (log-average of VTCN1, VISTA, TIGIT, PD1, LAG3, ADORA2A, IDO1/2, CTLA-4, PDCD1LG2 (PD-L2), CD274 (PD-L1), and HAVCR2) in COAD and READ, respectively. The Pearson’s rho (R) and statistical significance (*p*-value) are indicated in each graph. Loess regression (blue line) was used to diminish the noise of the variables during correlation analysis. **k** Kaplan-Meier curves depict the overall survival of COAD and READ patients after synergistic analysis for CTLA-4 and PD-L1 in these tumors. Both individual and simultaneous high levels of CTLA-4 and PD-L1 had a positive (though not statistically significant) effect on the patients' overall survival. On the reverse, simultaneous low expression of both genes led to a significant shift towards negative effect versus all other patients

Microsatellite-unstable (MSI) colorectal tumors were recently shown to over-express PD-L1, and thus be sensitive to immune checkpoint blockade with anti-PD-1 treatment [44, 45]. We speculated that MSI colorectal cancers might over-express several immune checkpoint molecules, other than PD-L1. To investigate this further, we calculated the expression of twelve known immune checkpoint molecules in MSI and MSS tumors and found significantly higher expression of all genes (except from *ADORA2* and *VTCN1*) in MSI COAD tumors. Higher expression was also scored for several checkpoints in MSI READ tumors (Fig. 4a-b). These data suggest that immune evasion is modulated in MSI-high tumors through the over-expression of more than one immune checkpoint molecules, thus providing tumor cells a selective pressure to escape from the cytotoxic T-cell/Th1 immune response.

**Fig. 5:**
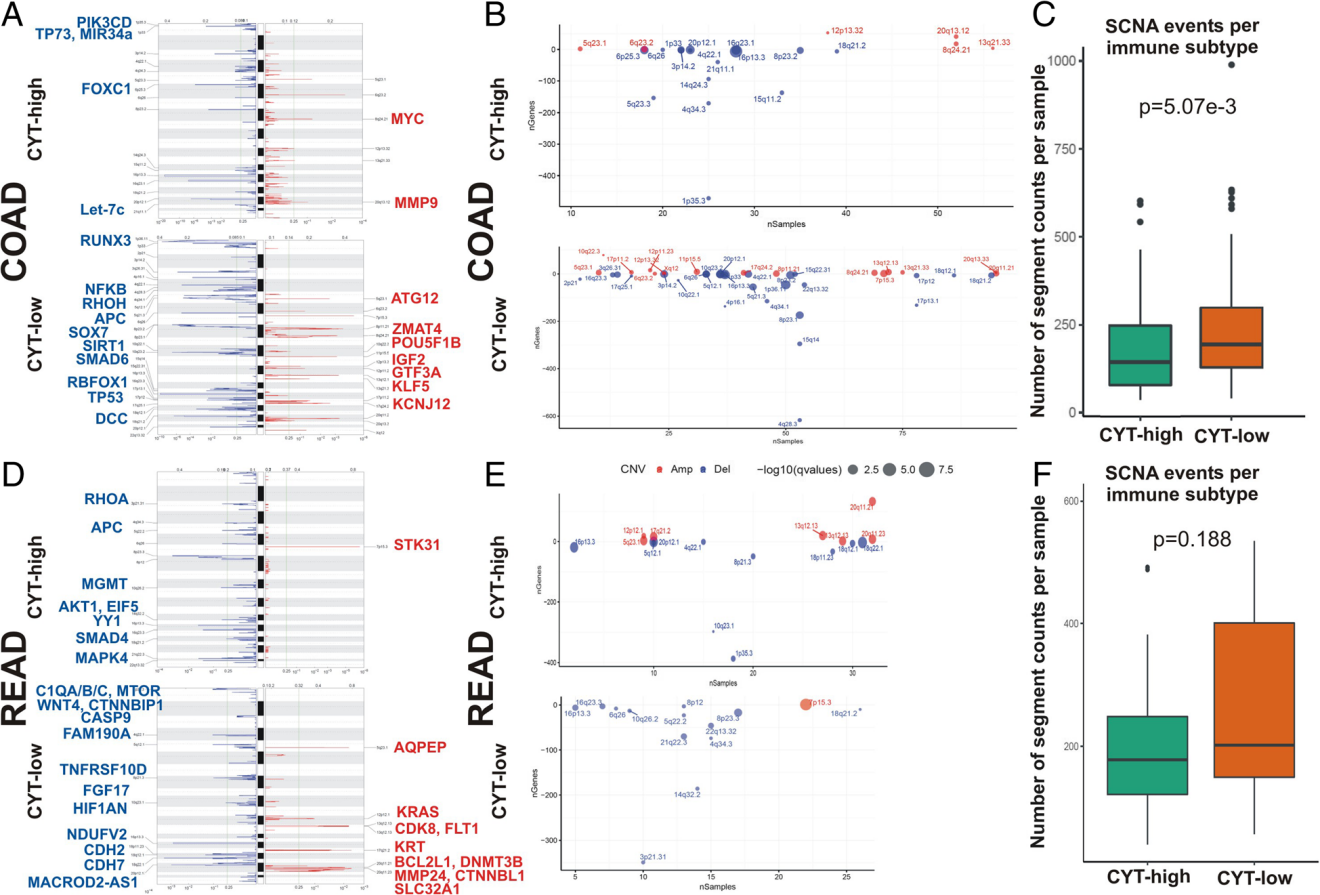
Assessed globally, GISTIC2.0 analysis identified a higher number of recurrent somatic copy number alterations (SCNA) in the CYT-low subset of colon (but not rectum) adenocarcinomas. **a** The genome plots depict the loci with significantly recurrent amplifications (red) and deletions (blue), among CYT-high and CYT-low COAD tumors, respectively. **b** Bubble plots display the summarized GISTIC results for each cytolytic subset in the COAD cohort. Significantly, amplified or deleted samples across the COAD dataset are depicted in red and blue colors, respectively. The x-axis depicts the number of samples and the y-axis depicts the number of genes. The size of the bubbles corresponds to the level of the calculated q-values (−log_10_(q-value)) for the aberrant genomic regions. **c** The number of the total recurrent SCNA events was significantly higher in the cytolytic-low COAD subset (*p* = 5.07e-3, Mann–Whitney). **d** The genome plots depict the loci with significantly recurrent amplifications (red) and deletions (blue), among CYT-high and CYT-low READ tumors, respectively. **e** Bubble plots display the summarized GISTIC results for each cytolytic subset in the READ cohort. Significantly, amplified or deleted samples across the READ dataset are depicted in red and blue colors, respectively. The x-axis depicts the number of samples and the y-axis depicts the number of genes. The size of the bubbles corresponds to the level of the calculated q-values (−log_10_(q-value)) for the aberrant genomic regions. **f** The number of the total recurrent SCNA events was higher in the cytolytic-low READ subset, but the difference was not statistically significant (*p* = 0.188, Mann–Whitney)

Microsatellite instability, along with chromosome instability (CIN), and chromosomal translocations, all lead to extensive copy number alterations in both human patients and genetically engineered mouse models [46–48]. We hypothesized that distinct events of genomic instability take place in each cytolytic subgroup in colorectal cancer. To verify this assumption, we performed GISTIC2.0 analysis for the tumors in each dataset and assessed copy number alterations between the two cytolytic subtypes. Assessed globally, our analysis identified a significantly higher number of recurrent somatic copy number alterations (SCNA) in the CYT-low subset of colon (but not rectal) adenocarcinomas (*p* = 5.07e-03). Cytolytic-low (but not high) colon adenocarcinomas had recurrent deletions at loci important in COAD, including 4q28.3 (*NFKB1*, *RHOH*), 5q21.3 (*APC*), 8p23.1 (*SOX7*), 10q22.1 (*SIRT1*), 16p13.3 (*RBFOX1*), 17p13.1 (*TP53*), 15q22.31 (*SMAD6*), 18q21.2 (*DCC*), and amplifications in 5q23.1 (*ATG12*), 8p11.21 (*ZMAT4*), 8q24.21 (*POU5F1B*), 11p15.5 (*IGF2*), 13q12.13 (*GTF3A*), 13q21.33 (*KLF5*), 17p11.2 (*KCNJ12*), among other alterations. Cytolytic-high colon tumors on the other hand, were characterized by recurrent deletions at loci 1p35.3 (*PIK3CD*, *TP73*, *miR34a*), 6p25.3 (*FOXC1*), and 21q11.1 (*Let*-*7c*), and amplifications at loci 8q24.21 (*MYC*) and 20q13.12 (*MMP9*) (Fig. 5a-c and Additional file 16: Table S7).

Likewise, CYT-low rectal tumors exhibited a higher number of SCNAs, but the difference with the CYT-high subgroup did not reach statistical significance. CYT-low (but not high) rectum tumors had recurrent deletions at loci 20p12.1 (*MACROD2-AS1*), 4q22.1 (*FAM190A*), 8p21.3 (*TNFRSF10D*, *FGF17*), 10q23.1 (*HIF1AN*), 18p11.23 (*NDUFV2*), 18q12.1 (*CDH2*), 18q22.1 (*CDH7*), and amplifications in 5q23.1 (*AQPEP*), 12p12.1 (*KRAS*), 13q12.13 (*FLT1*, *CDK8*), 17q21.2 (*KRT*), 20q11.21 (*BCL2L1*, *DNMT3B*, *MMP24*, *CTNNBL1*) and 20q11.23 (*SLC32A1*). CYT-high rectal tumors on the other hand, had recurrent deletions at loci 3p21.31 (*RHOA*), 5q22.2 (*APC*), 10q26.2 (*MGMT*), 14q32.2 (*AKT1*, *EIF5*, *YY1*), 18q21.2 (*SMAD4*, *MAPK4*), and a single amplification in 7p15.3 (*STK31*) (Fig. 5d-f and Additional file 17: Table S8). Taken together, these findings demonstrate that apart from somatic mutations, genomic instability due to distinct copy number alterations is characteristic for each cytolytic subgroup of colorectal adenocarcinomas.

Mutational analysis of tumor samples can be hampered due to tumor heterogeneity, which can reduce the ability to confidently detect SCNAs. To infer clonality, we calculated the number of different clusters within each tumor, along with the corresponding mean of each tumor’s variant allele frequency (VAF). Intra-tumor heterogeneity was measured calculating the width of each tumor’s VAF distribution (mutant-allele tumor heterogeneity, MATH scores). Higher MATH scores are found to be associated with poor outcome, and can be used as a proxy variable for survival analysis [49]. Highly variable intra-tumor heterogeneity among different samples was previously identified in rectal cancer [50].

MATH scores ranged from 7.24–78.24 in the COAD dataset and between 5.86–79.71 in the READ dataset. Tumor heterogeneity did not correlate with either total mutation load or total copy number events in the two CRC datasets. Moreover, there was no difference in the MATH scores between the two cytolytic subtypes (CYT-high vs –low COAD, 32.60 ± 13.38 vs 32.92 ± 15.51, *p* = 0.873; CYT-high vs –low READ, 31.46 ± 14.19 vs 32.74 ± .14, *p* = 0.716) suggesting that the observed differences in copy number and mutational load, were not a likely result of variable intra-tumor heterogeneity (Fig. 3i-j and Additional file 18: Table S9). Thus, distinct mutational and structural changes in the genome distinguish those CRCs with low versus high cytolytic activity.

### Correlation between the cytolytic index and neoepitope load

We then determined whether cytolytic activity is correlated with the neoepitope load in the two CRC datasets, as it has been widely suggested for cancers, in general [7]. Neoepitopes, derived from peptides encoded by somatic tumor mutations, and are thus not subject to central tolerance in the thymus, have been demonstrated to preferentially drive T-cell recognition of tumor cells [51]. To determine whether cytolytic activity is associated with the presence of neoepitopes, we determined the frequency of total missense mutations, and predicted those with potential to function as T-cell neoepitopes across the two datasets.

Antigen.garnish analysis revealed a significant correlation between the total mutation count per individual tumor and high cytolytic activity levels in COAD (Fig. 4c-d). Also, a significantly higher number of classically (CDN) and alternatively (ADN) defined neoepitopes (both MHC class I and II) was scored among CYT-high CRCs compared to CYT-low ones (Fig. 4e-f). Importantly, a high cytolytic index was correlated with MHC-I CDNs (*p* = 0.047, Pearson’s rho = 0.2) and MHC-II ADNs (*p* = 6.9e-05, Pearson’s rho = 0.39) in COAD tumors (Fig. 4g-h). Consistent with the findings from the overall mutation rate, MATH scores did not correlate with the number of CDNs and ADNs in CRC (Additional file 6: Figure S6). Taken together, these data suggest that cytolytic activity in CRC is driven by elevated mutation and/or neoepitope load.

### Cytokine and immune checkpoint expression patterns differ in colorectal tumors with high versus low cytolytic activity

The tumor microenvironment in colorectal cancer contains a rich cytokine milieu with both pro- and anti-inflammatory factors that can regulate tumorigenesis [52]. It is thus, expected that the expression of these cytokines and chemokines would be increased in CYT-high colorectal tumors. Balli et al. [30] recently reported increased expression of a series of pro- and anti-inflammatory cytokines and immune checkpoint molecules in CYT-high tumors. Consistent with these data, we found higher levels of a series of cytokines and chemokines (*CCR4*, *CCR5*, *CXCL9*, *CXCL10*, *CXCL11*, *CXCL13*, *C1QA*, *C1QB*, and *C1QC*), as well as of immune checkpoint molecules in both CRC datasets (Additional file 15: Table S6). Specifically, cytokines that were previously shown to correlate with the cytolytic index (*C1QA*, *C1QB*, *C1QC*, *CXCL9*, *CXCL10*, *CXCL11* and *CXCL13*) [7], were upregulated in CYT-high colorectal tumors. The expression of regulatory T cell (Treg) markers, such as *FOXP3* and *IL2RA*, was also significantly higher in CYT-high CRCs (Additional file 7: Figure S7).

CIBERSORT analysis showed that CYT-high CRCs were significantly related with high TIL levels, including CD4+ memory T cells, activated dendritic cells, and M2 macrophages, among others (*p* < 0.001) (Additional file 19: Table S10 and Additional file 8: Figure S8).

Finally, we assessed whether CYT-high colorectal cancers exhibit increased expression of immune checkpoint pathways. We investigated the expression levels of a series of inhibitory checkpoint molecules across COAD and READ patients, including *CD274* (*PD-L1*), *CTLA4*, *TIGIT*, *HAVCR2* (*TIM3*), *VISTA*, *PDCD1LG2* (*PD-L2*), *IDO1*, *IDO2*, *ADORA2A* (*A2AR*), *LAG3*, *PDCD1* (*PD-1*), *VISTA* (*C10orf54*), and *VTCN1* (*B7*-*H4*), among others. Importantly, we observed that higher cytolytic activity significantly correlated with high expression of at least five immune checkpoints (*CTLA-4*, *PD-1*, *PD-L1/2*, *LAG3* and *IDO1*) in CRC. This indicates that immune response in CYT-high colorectal tumors, similar to melanoma [53] and prostate cancer [28, 30] elicits multiple host and tumor mechanisms of immune suppression in the tumor microenvironment, other than the PD1 axis (Fig. 4i-j). Thus, our findings provide further evidence that a combinatorial targeting of such pathways may expand the clinical benefit for CRC patients.

Furthermore, using synergistic analysis for two of the most significant checkpoints, *CTLA-4* and *PD-L1*, we found that in COAD (but not in READ) tumors, both individual and simultaneous high levels of *CTLA*-*4* and *PD-L1* had a positive effect on the patients’ overall survival. On the reverse, simultaneous low expression of both genes led to a significant shift towards negative effect versus all other patients. These results provide evidence that high expression of both immune checkpoints affects synergistically the survival of colon cancer patients (Fig. 4k).

### Verification of gene expression using an independent cohort of colorectal cancer patients

**Fig. 6:**
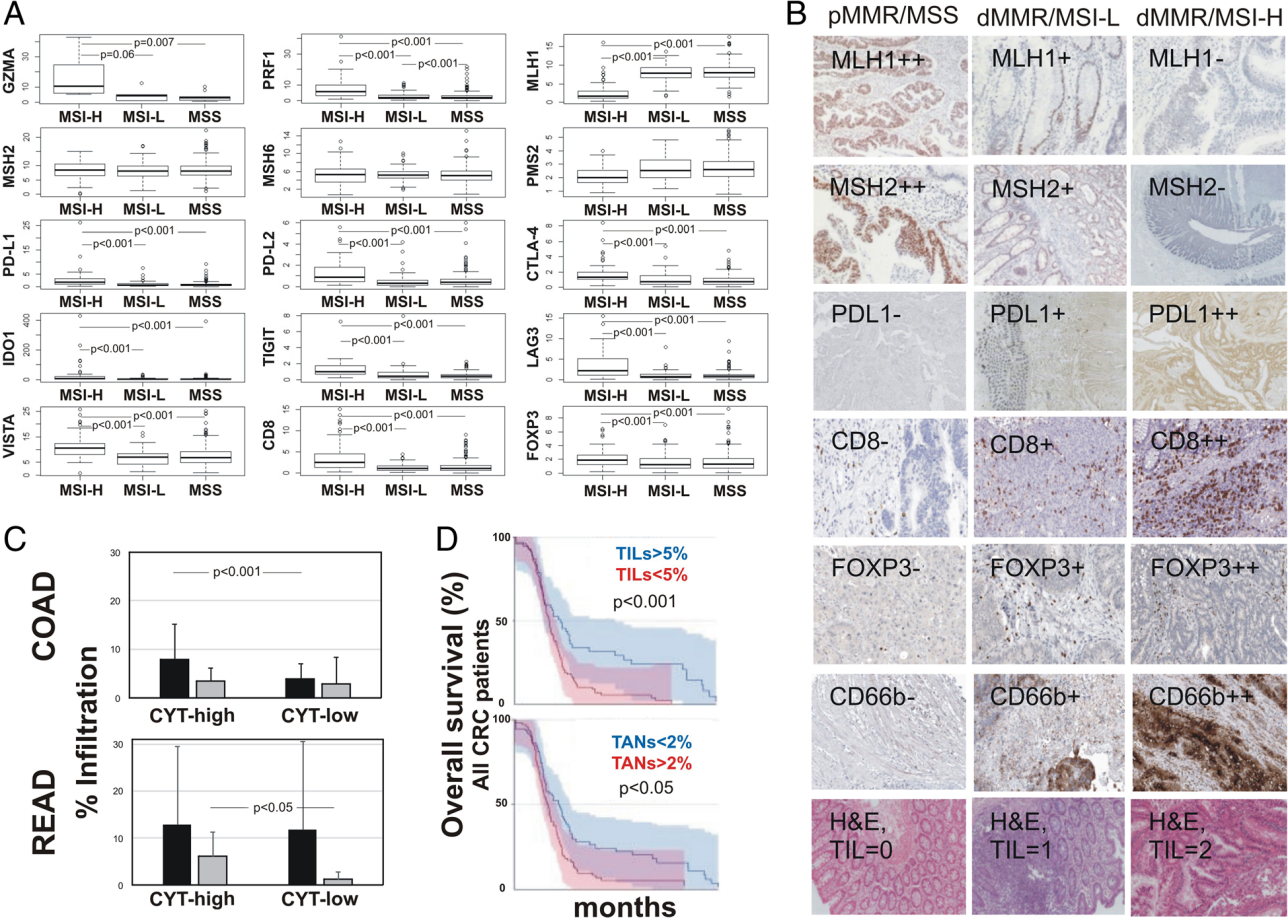
**a** Boxplots depicting expression levels of *GZMA*, *PRF1*, *CD274* (*PD-L1*), *PDCD1LG2* (*PD-L2*), *CTLA-4*, *IDO1*, *TIGIT*, *LAG3*, *VISTA*, *CD8*, *FOXP3*, *MLH1*, *MSH2*, *MSH6*, and *PMS2*, as measured by RT-qPCR in an independent cohort of 72 colorectal adenocarcinomas (12 MSI-H, 13 MSI-L and 47 MSS). **b** IHC staining for MLH1, MSH2, PD-L1, CD8, FOXP3, and CD66b in pMMR/MSS, dMMR/MSI-L and dMMR/MSI-H CRC patients. H&E, hematoxylin and eosin staining. **c** The percentage of tumor-infiltrating lymphocytes (TIL, black bars) and tumor-associated neutrophils (TAN, grey bars) in cytolytic high and low colon (COAD) and rectum (READ) adenocarcinomas, respectively. In the COAD dataset CYT-high tumors had higher TIL load; whereas in the READ dataset, CYT-high tumors had higher TAN load. **d** Overall survival between all CRCs with high or low TIL and TAN load, respectively. The patients were separated into high and low TIL or TAN groups, based on the median numbers of TILs and TANs (5% and 2%, respectively)

We further verified the expression of both cytolytic genes (*GZMA* and *PRF1*), MMR-related genes (*MLH1*, *MSH2*, *MSH6* and *PMS2*), immune checkpoint molecules (*PD-L1/2, CTLA-4, IDO1, TIGIT, LAG3, VISTA*), as well as that of *CD8* and *FOXP3* (Treg marker) in an independent cohort of 72 colorectal adenocarcinomas by RT-qPCR (Fig. 6a).

Furthermore, the protein expression of MLH1, MSH2 (MMR markers), PD-L1 (marker for T-cells, B-cells and tumor cells), CD8 (CTL marker), FOXP3 (Treg marker) and CD66b (TAN marker) was evaluated by IHC in FFPE tissue derived from the same patients in the cohort (47 pMMR/MSS, 13 dMMR/MSI-L and 12 dMMR/MSI-H colon adenocarcinomas). Medium or low PD-L1 expression was observed in 16/47 (~ 34%) pMMR/MSS and in 4/25 (16%) dMMR/MSI CRCs, whereas anti-PD-L1 staining was negative for the majority of the tumors. PD-L1+ microsatellite unstable CRCs also stained moderately for CD8 and weakly for FOXP3, indicating some infiltration of TILs and Tregs in these tumors. We also found higher CD66b + TAN infiltration in dMMR/MSI tumors (Fig. 6b).

To verify this assumption, we investigated whether the TIL load or that of TAN differs between the two cytolytic CRC subsets. TILs contained both stromal- and intratumoral-compartment lymphocytes, as previously defined [6, 54]. We found that CYT-high colon tumors had significantly higher TIL load compared to CYT-low tumors (*p* < 0.001), but the TAN load was equal between the two cytolytic subgroups. On the other hand, CYT-high rectal tumors had significantly higher TAN load (but not TILs) compared to the CYT-low tumors (*p* = 0.027) (Fig. 6c). Importantly, a high TIL load, and a low TAN load respectively, were significantly correlated with a better prognosis among all CRC patients (Fig. 6d). Supportive evidence on this finding also came from the analysis of patients’ survival in the TCGA database (Additional file 9: Figure S9).

## Discussion

Immunoediting has turned out to be progressively critical in appreciating the immune system’s ability to harness tumor growth and spread in several types of cancer [55]. In the present study, we implemented an extensive integrated analysis of the transcriptional and genetic landscape of colorectal cancer in the context of immune cytolytic activity. By stratifying colorectal cancer patients based on a validated cytolytic gene expression signature, we found a small subset with evidence of prominent T-cell reactivity.

Our data reveal a significant enrichment of CYT-high colon tumors for immune gene sets associated with activated CD8^+^, PD1^high^ T-cells. CYT-high tumors were also statistically enriched for gene sets including spleen interferon-producing DCs, amplification hotspots in various loci, and others. Regarding the first gene set, it has been shown that DCs may be endowed with a cytotoxic activity effective of killing tumor cells, apart from functioning as professional antigen presenting cells (APC) and controlling immune responses. For this reason they have been referred to as “interferon-producing killer dendritic cells (IKDCs)” [56]. These, are directly cytotoxic to NK targets, produce IFN-γ but not IFN-α, and appear to be functionally closer to NK cells than to plasmacytoid dendritic cells (pDCs) [57]. Furthermore, significantly amplified regions were previously associated with high cytolytic activity in CRC and other cancers, including those of the head and neck, cervix, stomach, and lung. Of major interest, these amplifications were found to include PDL1/2 and other immunosuppressive factors [7]. While tumor cells and TILs express such immune-suppressive ligands, our results corroborate that tumor-expressed ligands affect tumor fitness in the presence of cytolytic activity.

We also show that high CYT is significantly correlated with an increased mutational burden in the tumor, including a high load of predicted cancer neoepitopes. In agreement with previous reports [4, 7, 58], and contrasted to those in pancreatic cancer [30], we further show that high CYT significantly correlates with microsatellite unstable colon tumors. This increased mutational load among dMMR/MSI+ tumors was previously associated with prolonged progression-free survival (PFS) [44]. Importantly, our data reveal the existence of different cancer drivers between cytolytic-high and –low colorectal cancers. Recent studies have also established the direct determination of the tumor's mutational burden as a predictive biomarker for immunotherapy [59, 60]. However, it seems that not all dMMR/MSI+ CRC patients have also a high tumor mutational burden, since this was observed in the absence of dMMR/MSI, as well [61, 62].

We further showed that both colon and rectum MSI-H tumors are hypermutated and express numerous neoepitopes which elicit an immune response by TILs [21, 63]. Most of these mutant neoepitopes in MSI-H tumors render them sensitive to immune checkpoint blockade, regardless of the cancers’ tissue of origin [64]. The dMMR/MSI-H tumors are thought to possess greater TIL densities compared to pMMR/MSS tumors, due to the presence of a vast number of neoepitopes [44]. This explains why checkpoint inhibition in pMMR/MSS CRCs hasn’t been very successful yet; whereas dMMR/MSI-H tumors are more susceptible to PD-1/PD-L1 blockade.

We also showed that low cytolytic activity tracked with increased genomic structural variations, most notably prominent and recurrent *ATG12*, *KLF5* and *KCNJ12* amplifications and non-silent mutations and/or deletions in *APC*, *TP53*, *NFKB*, *DCC* and *SMAD6*, among others. Other distinct chromosomal aberrations were associated with CYT-high CRCs. These data point to an underappreciated link between genomic alterations and immune activation in colorectal cancer, suggesting that genomic structural variations implicated in the tumor's progression may also fundamentally influence de novo or therapeutic antitumor immune activation, independently of host immune factors.

Our data also reveal a significantly higher TIL density among CYT-high colon tumors, which was associated with improved overall survival of these patients. Cytolytic activity was shown to associate with improved survival in CRC due to increased immunity and cytolytic activity of T cells and M1 macrophages [6]. We also showed that high CYT levels are accompanied by upregulation of at least one immune-checkpoint molecule, indicating that immune responses in CYT-high tumors elicit immune suppression in the tumor microenvironment [28]. In addition, CYT-high rectal tumors had a higher TAN load, which was associated with a worse prognosis. This is in line with a recent report, according to which CRC patients with fewer CD66b + TANs showed statistically favorable survival rates [37]. We can thus, hypothesize that MSI+ tumors with increased cytolytic levels have a significant immunological response that is elicited by such neoepitopes. Nevertheless, such MSI+ CYT-high tumor cells are not effectively eliminated by the immune system, due to the increased levels of several immune-inhibitory checkpoint molecules, such as PD-L1, PD-L2, CTLA-4, LAG3, TIGIT, IDO1 and VISTA [21].

Finally, we found that CYT-high tumors exhibited increased expression of multiple immune checkpoints, including CTLA-4, PD-1, PD-L1/2, LAG3 and IDO1, the levels of which significantly correlated to the cytolytic index. Such an immune activation in response to high neoepitope load additionally suggests that a combinatorial targeting of more than one immune checkpoint pathways could be beneficial for hypermutated CRC patients. This is supported by recent clinical findings, based on which neoadjuvant immunotherapy with a combination of ipilimumab (anti-CTLA-4) plus nivolumab (anti-PD-1) in early stage dMMR/MSI and pMMR/MSS CRCs resulted in major pathological responses in 100% of the dMMR/MSI tumors, and did not compromise surgery [65]. Taken together, this new knowledge further emphasizes the high potential of neoadjuvant treatment with combinatorial targeting of more than one immune checkpoints.

Two clinical trials recommended the use of PD-1 blockade (pembrolizumab and nivolumab) for the treatment of metastatic CRC. In 2015, the KEYNOTE-16418 study showed that the MMR status can predict the clinical benefit of immune checkpoint blockade using pembrolizumab [44]. Of major interest, in the CHECKMATE-142 trial, nivolumab was used as second- or third-line treatment for patients with dMMR/MSI-H metastatic CRC and its combination with ipilimumab had comparatively better efficacy, and was shown to provide a promising new treatment option for metastatic CRC patients with dMMR/MSI+ tumors [25, 66].

The phase 2 study NCT01876511 is also on the way, investigating whether pembrolizumab is effective and safe in MSI+ CRC patients. Nevertheless, current success of immunotherapy is still limited to ~ 30% of MSI-H patients. Understanding why MSI-H tumors are responsive to immunotherapy will help develop better treatment options for all CRC patients. We propose that the CYT-high subgroup of these MSI-H tumors can be benefited to a higher percentage from such combinatorial immunotherapies. In line with our proposal, the combined use of immunotherapy with chemotherapy, ionizing radiation, and/or monoclonal antibodies, as well as the careful consideration of the right order of administering such combinations, is also proposed as a promising option that could complement the cancer-immunity cycle [67].

## Conclusions

In summary, our data support the utility of combining genomic and immune profiling for the comprehensive understanding of immune activation in CRC, an approach that can help guide the development of effective combinatorial immunotherapies in this disease. We provide proof of concept that the high mutational burden and cancer neoepitope load are the primary drivers of immune activity in CRC. In addition, our findings highlight the need to look beyond standard neoepitope-based strategies for immunotherapy in CRC and to focus further on other tumor-intrinsic features that render these tumors immune privileged. The upregulation of various immune checkpoints in CRC, as reflected by increased cytolytic levels, provides further evidence of preceding T-cell immunity, similar to melanoma and lung cancer. Finally, we propose that the CYT-high subset of dMMR/MSI colorectal cancer patients is more susceptible to combinatorial immune checkpoint blockade.

## Additional files

Additional file 1: **Figure S1.** The voom function was used to transform the read counts into log counts per million (CPMs) while taking into account the mean-variance relationship in the data [68]. The mean-variance trend plots below were made to detect any genes that possibly varied in the data, and filtering of the low counts was performed adequately. (JPG 1492 kb)

Additional file 2: **Figure S2.** Enrichment of selected immune-related gene sets in CYT-high colon (COAD) and rectum (READ) adenocarcinomas. **A.** Cytolytic-high tumors show increased enrichment of gene sets from activated, cytolytic CD8+ T-cell populations and PD1^high^ CD8 T-cells [38, 39]. **B.** Gene set variation analysis (GSVA) of known immune-related gene sets shows statistically significant increase in CYT-high colon and rectal tumors. (JPG 2337 kb)

Additional file 3: **Figure S3.** Gene set variation analysis (GSVA) of known immune-related gene sets (e.g., BIOCARTA_CTL_PATHWAY; TCYTOTOXIC_PATHWAY; TCRA_PATHWAY; THELPER_PATHWAY; PD1_SIGNALING; PRODUCING_DENDRITIC_CELL) showed statistically significant increase in CRCs identified as CYT-high, based on the expression of GZMA and PRF1. (JPG 1293 kb)

Additional file 4: **Figure S4.** *APC* and *KRAS* mutation types across the two CRC datasets and association with the cytolytic index, showing no statistically significant correlation between *APC* or *KRAS* mutations and CYT-high or -low subsets. (JPG 1232 kb)

Additional file 5: **Figure S5.** Top 25 mutually exclusive (< −1 log10 *p*-value) or co-occurring (> 1 log10 *p*-value) gene pairs in COAD and READ, using pair-wise Fisher’s exact test. The red arrow indicates significant mutual exclusivity between *BRAF* and *APC* in COAD. (JPG 530 kb)

Additional file 6: **Figure S6.** MATH scores did not correlate with the number of classically defined neoepitopes (CDN) or alternatively defined neoepitopes (ADN) neoepitopes in CRC. (JPG 474 kb)

Additional file 7: **Figure S7.** Expression of differentially expressed Treg markers in cytolytic subsets of colorectal cancer. (JPG 1825 kb)

Additional file 8: **Figure S8.** CIBERSORT analysis results (in silico flow cytometry) depict the fractional representation of 22 hematopoietic cell types present in the gene expression profile of each cytolytic subset in colon (COAD) and rectal (READ) cancers, respectively. Columns represent cell types from the signature genes file and rows represent the deconvolution results for each tumor sample within each cytolytic subgroup. Filtering was set at *p* = 0.05 with 1000 permutations during analysis. All results are reported as relative fractions normalized to 1 across all cell subsets. *P*-value: Statistical significance of the deconvolution result across all cell subsets; useful for filtering out results with a poor “goodness of fit”. Correlation: Pearson’s correlation coefficient (R), generated from comparing the original mixture with the estimated mixture, the latter of which is calculated using imputed cell fractions and corresponding expression profiles from the signature genes file. Of note, the correlation is restricted to signature genes. RMSE: Root mean squared error between the original mixture and the imputed mixture, restricted to genes in the signature gene file. (JPG 1521 kb)

Additional file 9: **Figure S9.** Overall survival between CRCs with high and low load of tumor-assocated neutrophils (TAN load), using combined CRC samples from the TCGA-COAD and READ datasets, respectively. The patients were separated into high and low TAN load, based on the median number of TANs. (JPG 19 kb)

Additional file 10: **Table S1.** The CRC samples within each dataset (COAD and READ), along with their clinical information and cytolytic levels (CYT). (XLSX 9 kb)

Additional file 11: **Table S2.** Microsatellite instability status of all colorectal cancer samples within each TCGA dataset (COAD and READ). The presence of missense mutations in the MMR genes MLH1, MLH3, MSH2, MSH3, MSH4, MSH5, MSH6, PMS1 and PMS2 was used to stratify tumor samples to microsatellite unstable (MSI) or stable (MSS), respectively. (XLSX 16 kb)

Additional file 12: **Table S3.** Primer sequences used for RT-qPCR. (XLSX 4938 kb)

Additional file 13: **Table S4.** Human Protein Atlas (HPA)-derived protein expression analysis shows that in colorectal cancer GZMA is lowly expressed (3/11, 27%) or not detected (8/11, 72.7%) and PRF1 protein is not detected in any of the 12 colorectal cancer samples. (XLSX 71455 kb)

Additional file 14: **Table S5.** Patient IDs and cytolytic activity levels (CYT) for CYT-high and CYT-low cytolytic subgroups of COAD and READ tumors. (XLSX 27546 kb)

Additional file 15: **Table S6.** Top-upregulated genes in the cytolytic-high (CYT-high) COAD and READ tumors, against their cytolytic-low (CYT-low) counterparts. (XLSX 9165 kb)

Additional file 16: **Table S7.** GISTIC (v2.0.22) analysis results for colon cancers (COAD). GISTIC was used to detect recurrent somatic copy number alterations (SCNA) in CYT-high and -low colon cancers, using MutSigCV (v1) from the Broad Institute’s GenePattern. Each SCNA was assigned a G-score indicative of its amplitude and occurrence across samples. SCNAs in each COAD sample within each cytolytic subgroup were counted by taking the sum of segment mean changes ≥ 0.6 and ≤ − 0.4 between somatic and normal samples. Significantly amplified or deleted genomic regions in each cytolytic subgroup with FDR < 0.25 were considered significant. (XLSX 95 kb)

Additional file 17: **Table S8.** GISTIC (v2.0.22) analysis results for rectal cancers (READ). GISTIC was used to detect recurrent somatic copy number alterations (SCNA) in CYT-high and -low rectum cancers, using MutSigCV (v1) from the Broad Institute’s GenePattern. Each SCNA was assigned a G-score indicative of its amplitude and occurrence across samples. SCNAs in each RED sample within each cytolytic subgroup were counted by taking the sum of segment mean changes ≥ 0.6 and ≤ − 0.4 between somatic and normal samples. Significantly amplified or deleted genomic regions in each cytolytic subgroup with FDR < 0.25 were considered significant. (XLSX 75 kb)

Additional file 18: **Table S9.** Tumor heterogeneity was inferred using “maftools”, with which we clustered variant allele frequencies (VAF). We also measured the extent of heterogeneity in terms of a numerical value. Mutant-Allele Tumor Heterogeneity (MATH) score is a simple quantitative measure of intra-tumor heterogeneity, which calculates the width of the VAF distribution. (XLSX 86 kb)

Additional file 19: **Table S10.** CIBERSORT analysis results depict the fractional representations of 22 hematopoietic cell types present in the gene expression profile of each cytolytic subset in colon (COAD) and rectal (READ) cancers, respectively. Columns represent cell types from the signature genes file and rows represent deconvolution results for each tumor sample in each cytolytic subgroup. Filtering was set at *p* = 0.05 with 1000 permutations during analysis. All results are reported as relative fractions normalized to 1 across all cell subsets. *P*-value: Statistical significance of the deconvolution result across all cell subsets; useful for filtering out results with a poor “goodness of fit”. Correlation: Pearson’s correlation coefficient (R), generated from comparing the original mixture with the estimated mixture, the latter of which is calculated using imputed cell fractions and corresponding expression profiles from the signature genes file. Of note, the correlation is restricted to signature genes. RMSE: Root mean squared error between the original mixture and the imputed mixture, restricted to genes in the signature gene file. (XLSX 10 kb)

## Data Availability

All data generated or analysed during this study are included in this published article [and its supplementary information files].

## Abbreviations

ADN: Alternatively defined neoepitope
CDN: Classically defined neoepitope
CMSs: Consensus molecular subtypes
CNA: Copy number alteration
COAD: Colon adenocarcinoma
CRC: Colorectal cancer
CTL: Cytotoxic T cells
CYT: Immune cytolytic activity
dMMR: Defective mismatch repair system
FFPE: Formalin-Fixed Paraffin-Embedded
GSVA: Gene set variation analysis
GZMA: Granzyme A
HPA: Human Protein Atlas
IHC: Immunohistochemistry
KS: Kolmogorov–Smirnov
MATH: Mutant-allele tumor heterogeneity
MSI: Microsatellite Instability
MSigDB: Molecular Signatures Database
NK cells: Natural killer cells
pMMR: Proficient mismatch repair system
PRF1: Perforin-1
READ: Rectum adenocarcinoma
SMGs: Significantly mutated genes
TCGA: The Cancer Genome Atlas
VAF: Variant allele frequency
