# Supplementary figures and images for: Cytolytic activity correlates with the mutational burden and deregulated expression of immune checkpoints in colorectal cancer
Source: J Exp Clin Cancer Res. 2019 Aug 20;38:364. doi: 10.1186/s13046-019-1372-z (PMC6701076; doi:10.1186/s13046-019-1372-z)

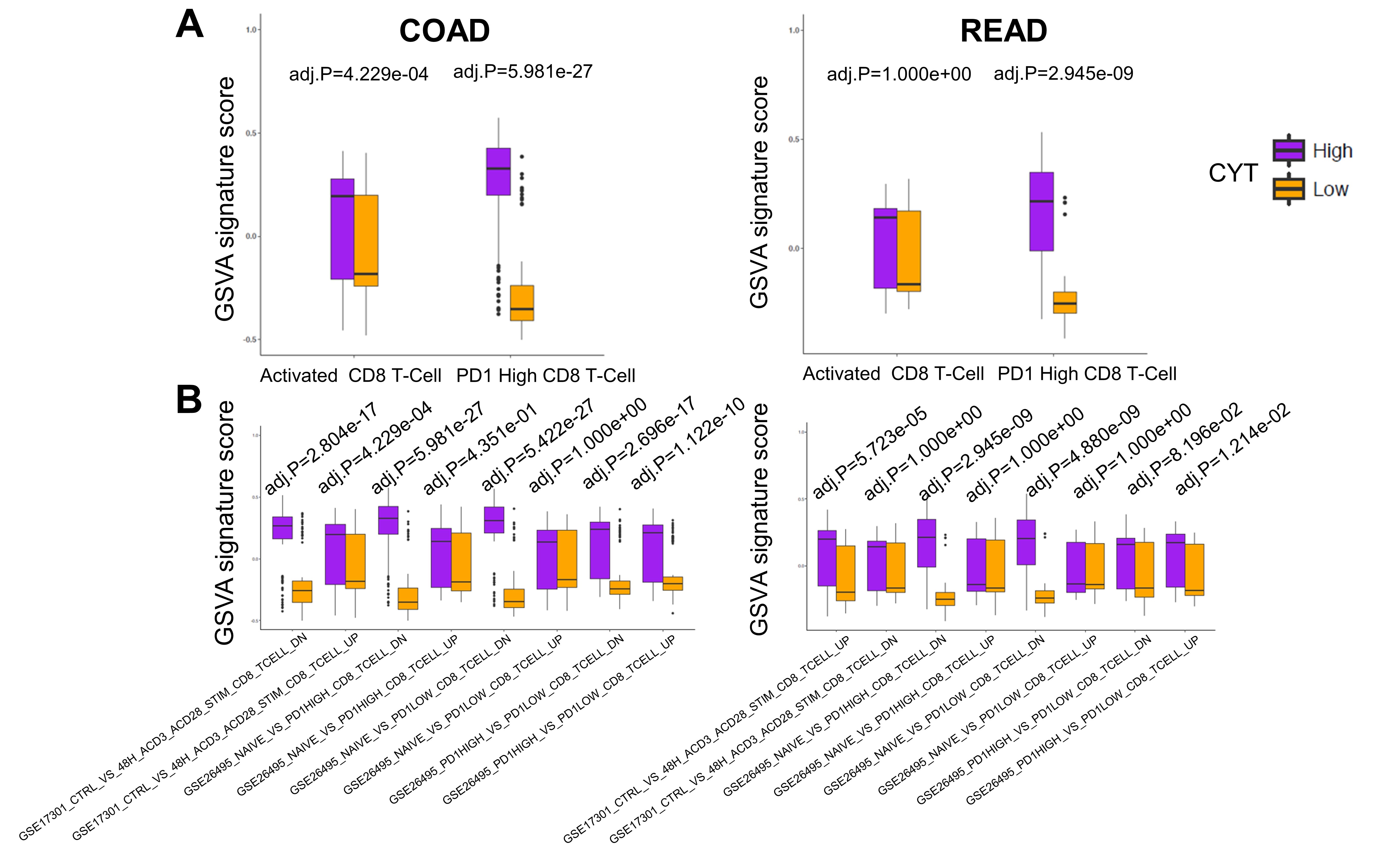

Supplement: Supplementary file 1 — Figure S1. The voom function was used to transform the read counts into log counts per million (CPMs) while taking into account the mean-variance relationship in the data [68]. The mean-variance trend plots below were made to detect any genes that possibly varied in the data, and filtering of the low counts was performed adequately. (JPG 1492 kb) [file 13046_2019_1372_MOESM1_ESM.jpg]

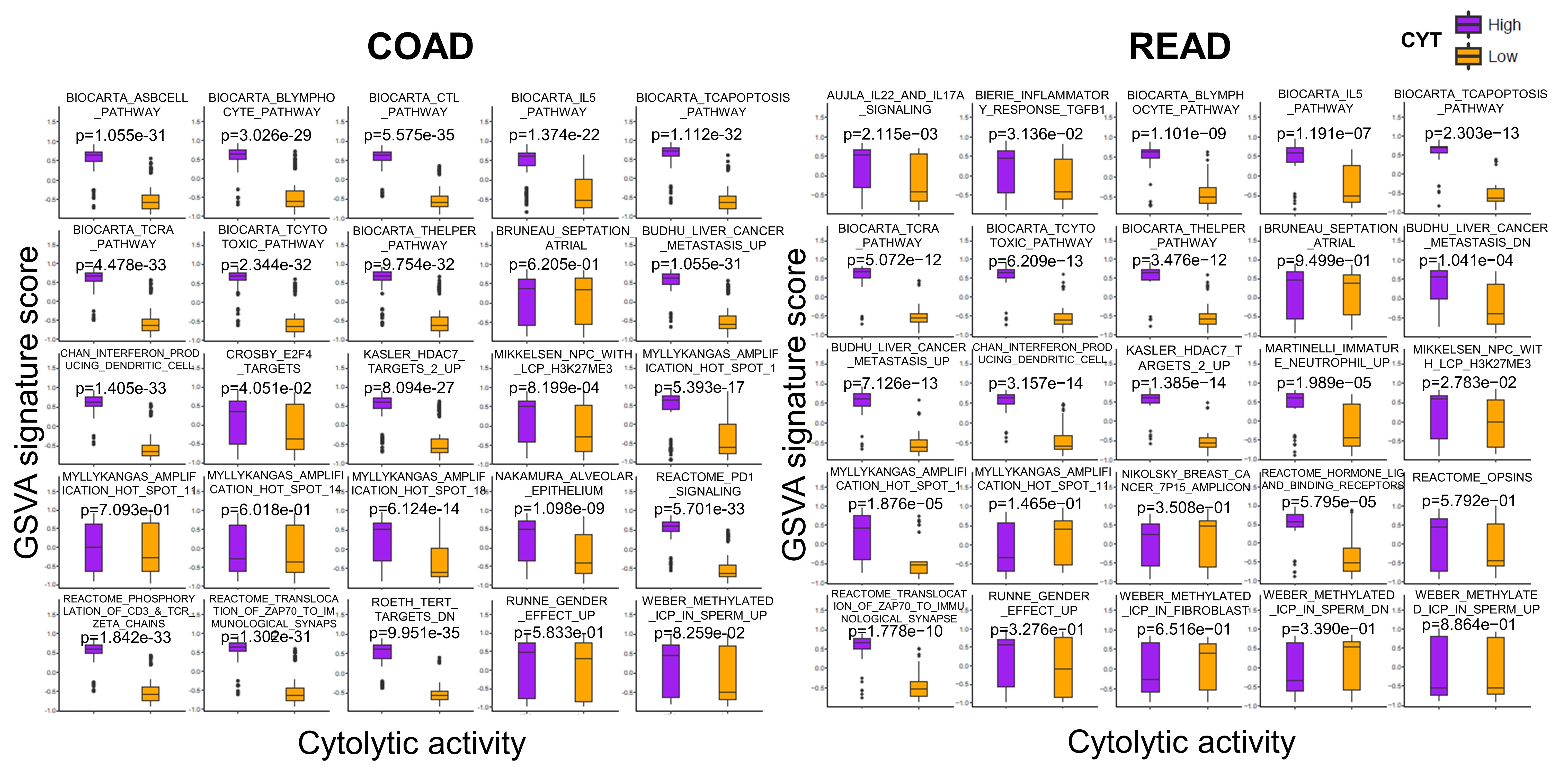

Supplement: Supplementary file 2 — Figure S2. Enrichment of selected immune-related gene sets in CYT-high colon (COAD) and rectum (READ) adenocarcinomas. A. Cytolytic-high tumors show increased enrichment of gene sets from activated, cytolytic CD8+ T-cell populations and PD1high CD8 T-cells [38, 39]. B. Gene set variation analysis (GSVA) of known immune-related gene sets shows statistically significant increase in CYT-high colon and rectal tumors. (JPG 2337 kb) [file 13046_2019_1372_MOESM2_ESM.jpg]

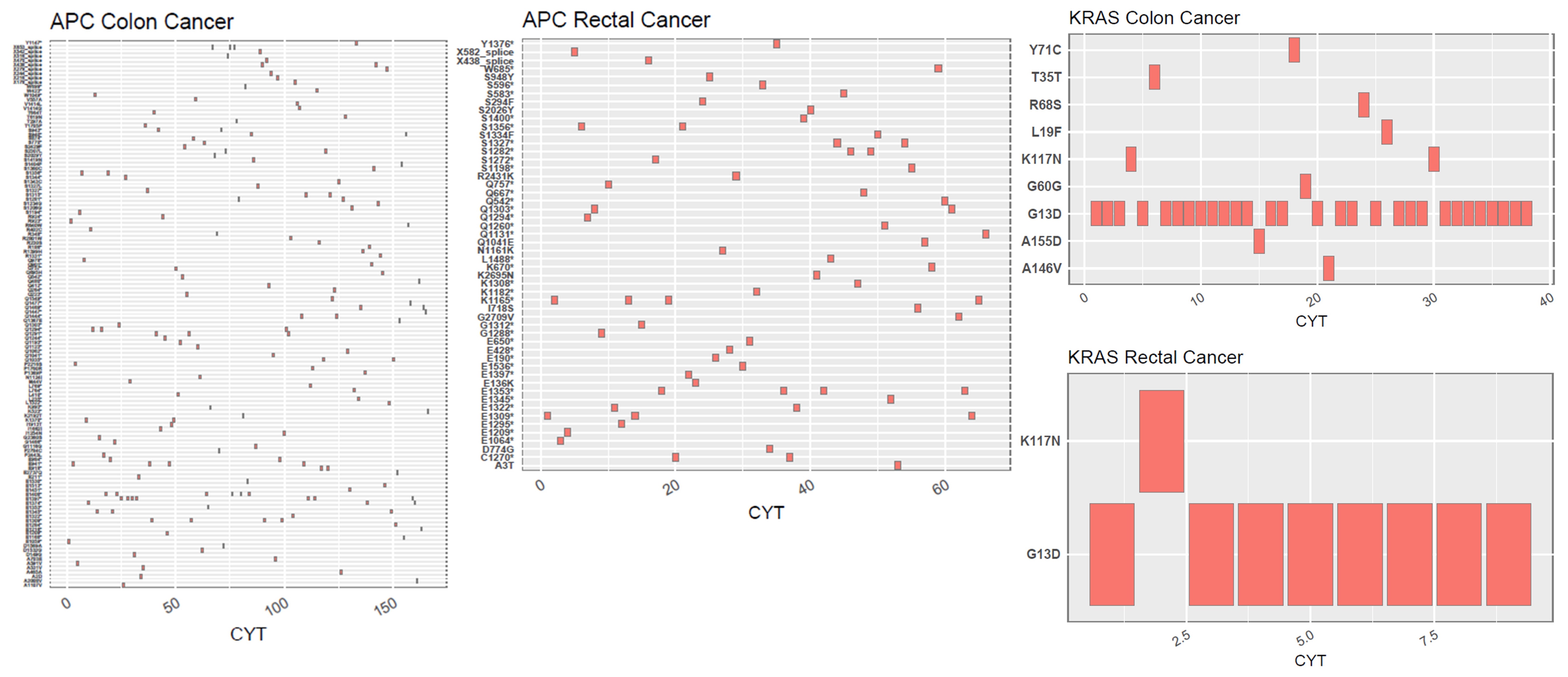

Supplement: Supplementary file 3 — Figure S3. Gene set variation analysis (GSVA) of known immune-related gene sets (e.g., BIOCARTA_CTL_PATHWAY; TCYTOTOXIC_PATHWAY; TCRA_PATHWAY; THELPER_PATHWAY; PD1_SIGNALING; PRODUCING_DENDRITIC_CELL) showed statistically significant increase in CRCs identified as CYT-high, based on the expression of GZMA and PRF1. (JPG 1293 kb) [file 13046_2019_1372_MOESM3_ESM.jpg]

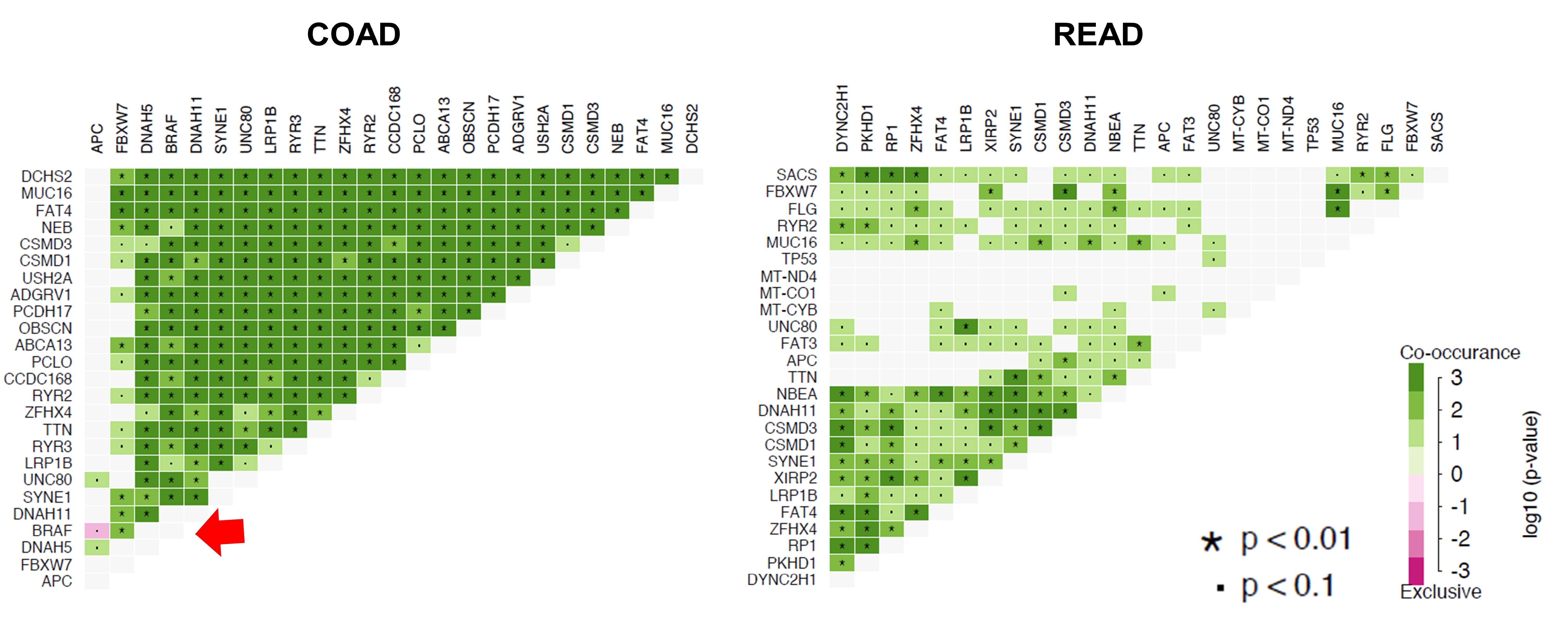

Supplement: Supplementary file 4 — Figure S4. APC and KRAS mutation types across the two CRC datasets and association with the cytolytic index, showing no statistically significant correlation between APC or KRAS mutations and CYT-high or -low subsets. (JPG 1232 kb) [file 13046_2019_1372_MOESM4_ESM.jpg]

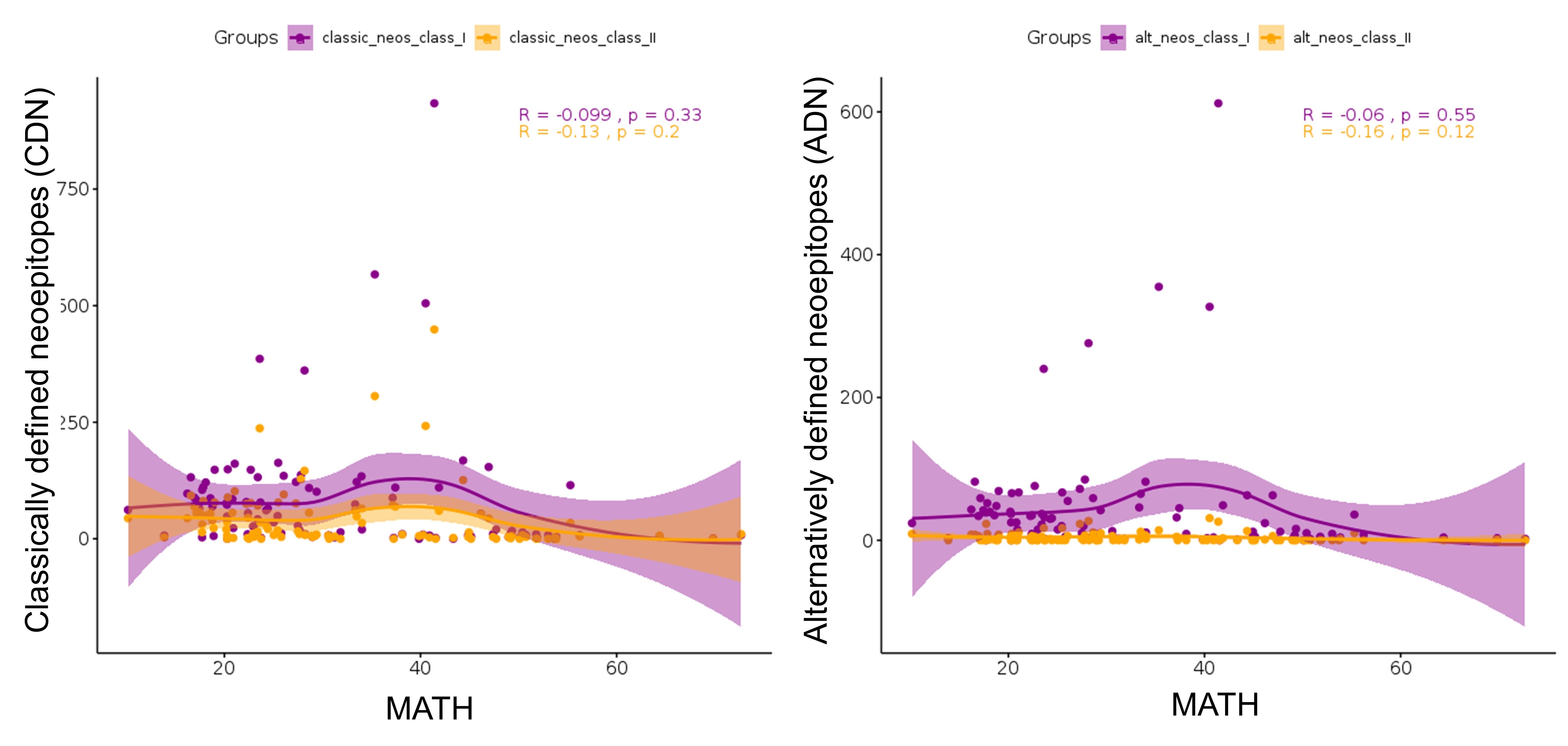

Supplement: Supplementary file 5 — Figure S5. Top 25 mutually exclusive (< −1 log10 p-value) or co-occurring (> 1 log10 p-value) gene pairs in COAD and READ, using pair-wise Fisher’s exact test. The red arrow indicates significant mutual exclusivity between BRAF and APC in COAD. (JPG 530 kb) [file 13046_2019_1372_MOESM5_ESM.jpg]

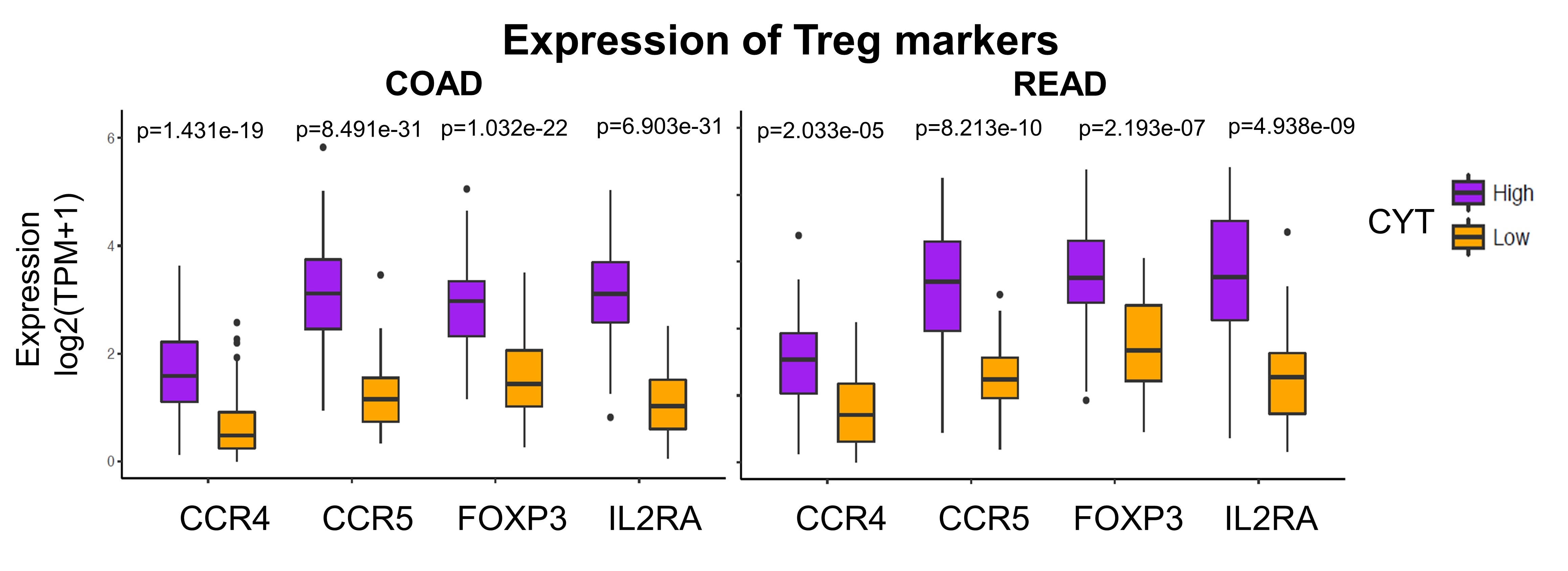

Supplement: Supplementary file 6 — Figure S6. MATH scores did not correlate with the number of classically defined neoepitopes (CDN) or alternatively defined neoepitopes (ADN) neoepitopes in CRC. (JPG 474 kb) [file 13046_2019_1372_MOESM6_ESM.jpg]

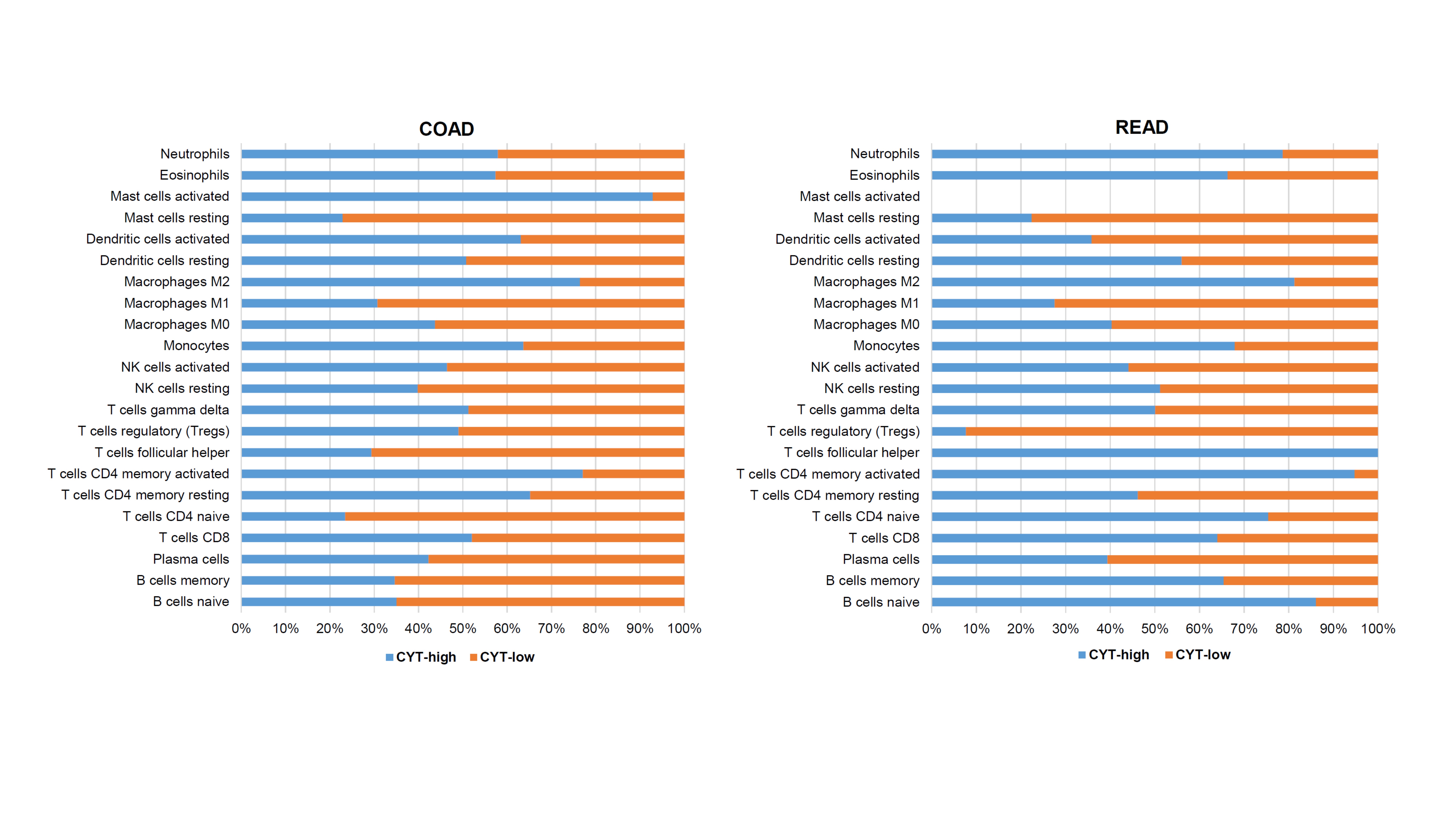

Supplement: Supplementary file 7 — Figure S7. Expression of differentially expressed Treg markers in cytolytic subsets of colorectal cancer. (JPG 1825 kb) [file 13046_2019_1372_MOESM7_ESM.jpg]

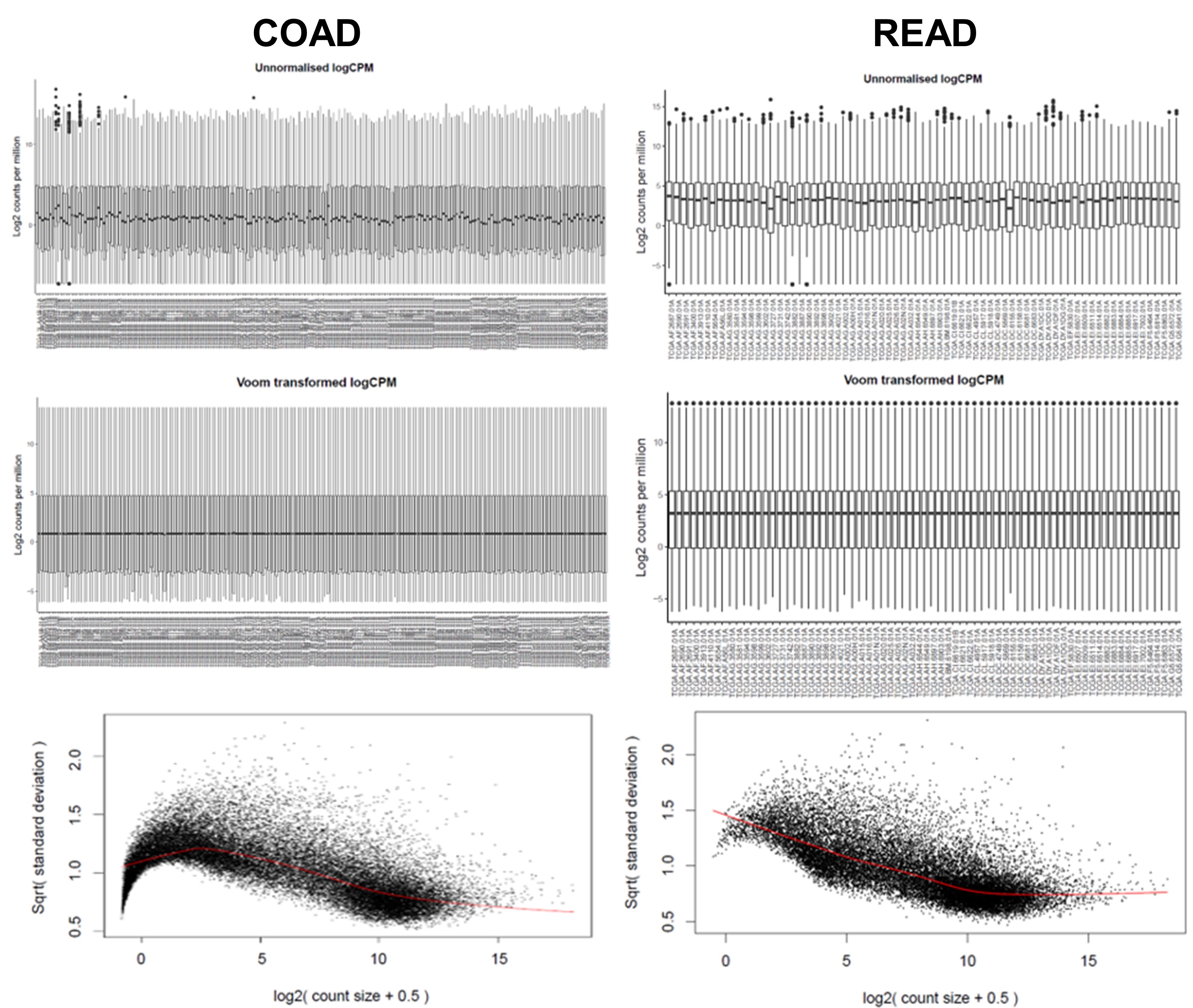

Supplement: Supplementary file 8 — Figure S8. CIBERSORT analysis results (in silico flow cytometry) depict the fractional representation of 22 hematopoietic cell types present in the gene expression profile of each cytolytic subset in colon (COAD) and rectal (READ) cancers, respectively. Columns represent cell types from the signature genes file and rows represent the deconvolution results for each tumor sample within each cytolytic subgroup. Filtering was set at p = 0.05 with 1000 permutations during analysis. All results are reported as relative fractions normalized to 1 across all cell subsets. P-value: Statistical significance of the deconvolution result across all cell subsets; useful for filtering out results with a poor “goodness of fit”. Correlation: Pearson’s correlation coefficient (R), generated from comparing the original mixture with the estimated mixture, the latter of which is calculated using imputed cell fractions and corresponding expression profiles from the signature genes file. Of note, the correlation is restricted to signature genes. RMSE: Root mean squared error between the original mixture and the imputed mixture, restricted to genes in the signature gene file. (JPG 1521 kb) [file 13046_2019_1372_MOESM8_ESM.jpg]

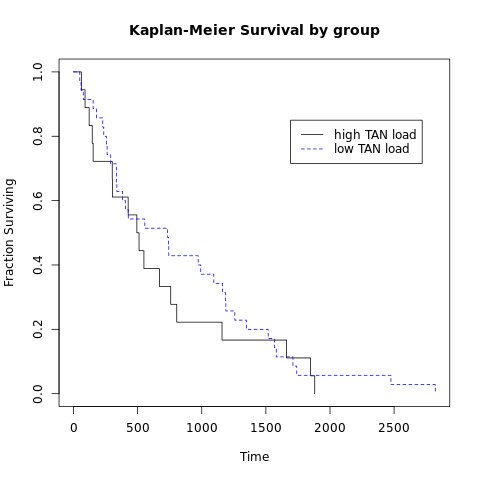

Supplement: Supplementary file 9 — Figure S9. Overall survival between CRCs with high and low load of tumor-assocated neutrophils (TAN load), using combined CRC samples from the TCGA-COAD and READ datasets, respectively. The patients were separated into high and low TAN load, based on the median number of TANs. (JPG 19 kb) [file 13046_2019_1372_MOESM9_ESM.jpg]
